# Automated Explainable Multidimensional Deep Learning Platform of Retinal Images for Retinopathy of Prematurity Screening

**DOI:** 10.1001/jamanetworkopen.2021.8758

**Published:** 2021-05-05

**Authors:** Ji Wang, Jie Ji, Mingzhi Zhang, Jian-Wei Lin, Guihua Zhang, Weifen Gong, Ling-Ping Cen, Yamei Lu, Xuelin Huang, Dingguo Huang, Taiping Li, Tsz Kin Ng, Chi Pui Pang

**Affiliations:** 1 Joint Shantou International Eye Center of Shantou University, the Chinese University of Hong Kong, Shantou, Guangdong, China; 2 Network and Information Center, Shantou University, Shantou, Guangdong, China; 3 XuanShi Med Tech (Shanghai) Company Limited, Shanghai, China; 4 Department of Ophthalmology, The Sixth Affiliated Hospital of Guangzhou Medical University, Qingyuan People’s Hospital, Qingyuan, Guangdong, China; 5 Department of Ophthalmology, Guangdong Women and Children Hospital, Guangzhou, Guangdong, China; 6 Shantou University Medical College, Shantou, Guangdong, China; 7 Department of Ophthalmology and Visual Sciences, The Chinese University of Hong Kong, Hong Kong, China

## Abstract

**Importance:**

A retinopathy of prematurity (ROP) diagnosis currently relies on indirect ophthalmoscopy assessed by experienced ophthalmologists. A deep learning algorithm based on retinal images may facilitate early detection and timely treatment of ROP to improve visual outcomes.

**Objective:**

To develop a retinal image–based, multidimensional, automated, deep learning platform for ROP screening and validate its performance accuracy.

**Design, Setting, and Participants:**

A total of 14 108 eyes of 8652 preterm infants who received ROP screening from 4 centers from November 4, 2010, to November 14, 2019, were included, and a total of 52 249 retinal images were randomly split into training, validation, and test sets. Four main dimensional independent classifiers were developed, including image quality, any stage of ROP, intraocular hemorrhage, and preplus/plus disease. Referral-warranted ROP was automatically generated by integrating the results of 4 classifiers at the image, eye, and patient levels. DeepSHAP, a method based on DeepLIFT and Shapley values (solution concepts in cooperative game theory), was adopted as the heat map technology to explain the predictions. The performance of the platform was further validated as compared with that of the experienced ROP experts. Data were analyzed from February 12, 2020, to June 24, 2020.

**Exposure:**

A deep learning algorithm.

**Main Outcomes and Measures:**

The performance of each classifier included true negative, false positive, false negative, true positive, F1 score, sensitivity, specificity, receiver operating characteristic, area under curve (AUC), and Cohen unweighted κ.

**Results:**

A total of 14 108 eyes of 8652 preterm infants (mean [SD] gestational age, 32.9 [3.1] weeks; 4818 boys [60.4%] of 7973 with known sex) received ROP screening. The performance of all classifiers achieved an F1 score of 0.718 to 0.981, a sensitivity of 0.918 to 0.982, a specificity of 0.949 to 0.992, and an AUC of 0.983 to 0.998, whereas that of the referral system achieved an F1 score of 0.898 to 0.956, a sensitivity of 0.981 to 0.986, a specificity of 0.939 to 0.974, and an AUC of 0.9901 to 0.9956. Fine-grained and class-discriminative heat maps were generated by DeepSHAP in real time. The platform achieved a Cohen unweighted κ of 0.86 to 0.98 compared with a Cohen κ of 0.93 to 0.98 by the ROP experts.

**Conclusions and Relevance:**

In this diagnostic study, an automated ROP screening platform was able to identify and classify multidimensional pathologic lesions in the retinal images. This platform may be able to assist routine ROP screening in general and children hospitals.

## Key Points

**Question:**

Can deep learning algorithms achieve a performance comparable with that of ophthalmologists on multidimensional identification of retinopathy of prematurity (ROP) using wide-field retinal images?

**Findings:**

In this diagnostic study of 14 108 eyes of 8652 preterm infants, a deep learning–based ROP screening platform could identify retinal images using 5 classifiers, including image quality, stages of ROP, intraocular hemorrhage, preplus/plus disease, and posterior retina. The platform achieved an area under the curve of 0.983 to 0.998, and the referral system achieved an area under the curve of 0.9901 to 0.9956; the platform achieved a Cohen κ of 0.86 to 0.98 compared with 0.93 to 0.98 by the ROP experts.

**Meaning:**

Results suggest that a deep learning platform could identify and classify multidimensional ROP pathological lesions in retinal images with high accuracy and could be suitable for routine ROP screening in general and children’s hospitals.

## Teaser

This diagnostic study uses data from the eye examinations of preterm infants to assess whether a deep learning algorithm can perform at a level comparable with that of ophthalmologists in identifying retinopathy of prematurity on wide-field retinal images.

## Introduction

Retinopathy of prematurity (ROP) is a leading cause of visual impairment and irreversible blindness of children worldwide, mainly affecting preterm infants with extremely low birth weight and those who are small for gestational age. Approximately 1.2% (184 700 of 1 4900 000) of preterm infants worldwide have been estimated to have ROP, among which 30 000 preterm infants have permanent visual impairment.^1^ Poor visual outcomes from ROP can be largely avoided if ROP is detected early and treated appropriately.^2^

In clinical scenarios, 3 ROP-related features (the stages of ROP and preplus/plus disease [considered specific features] and intraocular hemorrhage [considered a risk-indicative feature])^3,4,5^ have been adopted in ROP detection among preterm infants. According to the International Classification of Retinopathy of Prematurity, stages 1 to 5 of ROP are defined as abnormal response of immature vasculature in the retina, with increasing severity from stage 1 to 5. Preplus/plus disease is a continuum of abnormal changes with dilatation and tortuosity of posterior pole retinal vessels, indicating the need for intensive observation or treatment.^6,7^ In addition, intraocular hemorrhage is reported as a frequent predictor of the presence of ROP and poor outcomes in preterm infants.^8,9^ The standard method for ROP diagnosis relies on indirect ophthalmoscopy, which requires assessments performed by experienced ophthalmologists. In remote areas and places where ROP expertise is not readily available, a delayed or missed diagnosis of ROP can lead to vision loss.^10^ The development of an automated ROP screening platform that can meet the diagnostic criteria should facilitate timely treatment for patients.

Deep learning (DL) algorithms, especially convolutional neural networks (CNNs), have been widely applied in medical image analysis for different diseases, including glaucoma, intracranial hemorrhage, and lung cancers.^11,12^ Image-based automated ROP screening systems using deep CNNs have also been developed. Specifically, Hu et al^13^ focused on the stages of ROP detection at the image level and used the Guided Backpropagation^14^ algorithm to visualize the lesion based on a data set of 5511 retinal images; Brown et al^15^ developed a classifier for the differentiation of normal from the preplus and plus diseases using images of the posterior retina; and Wang et al^16^ developed a model of 2 deep CNN networks for classifying ROP into gradations of normal, minor, and severe. Their model adopted the multi-instance learning^17^ method and only generated eye-level results. However, these studies focused only on a single-dimensional classifier. Herein, we aimed to develop an automated multidimensional platform for ROP detection and screening using retinal images.

In this study, to address the previously mentioned problems, we developed an automated classification system covering 4 independent main classifiers (image quality, any stage of ROP, intraocular hemorrhage, and preplus/plus disease) and 1 auxiliary parameter (the posterior retina). We also developed an algorithm for the referral recommendation by integrating different outcomes from multiple dimensional analyses. The performance of our automated platform was further validated and compared with that of the ROP experts. This cloud-based platform was opened for external validation.

## Methods

This diagnostic study, conducted from September 1, 2018, to June 24, 2020, was performed in compliance with the Declaration of Helsinki^18^ and approved by the Human Medical Ethics Committee of Joint Shantou International Eye Center of Shantou University and the Chinese University of Hong Kong. Written informed consents were waived because the retinal images used for platform development were deidentified for personal information. This study followed the Standards for Reporting of Diagnostic Accuracy (STARD) reporting guideline.

### Data Sets

Retinal images of infants taken by corneal contact retinal cameras RetCam II or III (Clarity Medical Systems) for ROP screening were collected from 4 centers in southern China: Joint Shantou International Eye Center of Shantou University and the Chinese University of Hong Kong (JSIEC), Guangdong Women and Children Hospital in Yuexiu branch (Yuexiu) and Panyu branch (Panyu), and the Sixth Affiliated Hospital of Guangzhou Medical University and Qingyuan People’s Hospital (Qingyuan) (eFigure 1 in the Supplement). All retinal images including those of the normal fundus or those displaying any ROP were included. Exclusion criteria comprised (1) nonfundus photos or fundus photos taken by imaging devices other than RetCam; (2) infants with other ocular diseases, eg, congenital cataract, retinoblastoma, or persistent hyperplastic primary vitreous; and (3) any images with disagreeing labels.

### Image Labeling and Grading

Binary classification was used to categorize each of the 5 dimensions related to ROP retinal image diagnosis; this system met the requirements of the screening application, including (1) image quality (defined as gradable or ungradable; ungradable images were defined as those of poor quality with significant blur, darkness, defocus, poor exposure, or numerous artifacts that could not be identified; the remaining were classified as gradable), (2) any stage of ROP (defined as any stage or nonstage; any stage was assigned to images with any stage of ROP identified, whereas nonstage was assigned to those without any stage of ROP), (3) intraocular hemorrhage (defined as hemorrhage or nonhemorrhage; hemorrhage was assigned to the images with any identifiable hemorrhage), (4) preplus/plus disease (defined as preplus/plus or non–preplus/plus; preplus/plus described a spectrum of posterior retinal vessel abnormalities including venous dilation and arteriolar tortuosity, whereas non–preplus/plus described normal vessels in the posterior retina), and (5) posterior retina (defined as posterior or nonposterior). In order to accurately identify preplus/plus disease, the region of the posterior retina had to be defined. In this study, the posterior retina was defined as a circular area centered at the optic disc with a radius 3 times the diameter of the optic disc. Any portion of the images within this predefined area were classified as within the posterior pole.

The ground truth (criterion-standard) labels were determined by a group of ophthalmologists. The graders were trained according to our previously published protocol.^19^ Briefly, 2 trained junior ophthalmologists labeled independently, and the images with disagreeing labels were submitted to a senior ophthalmologist. If the decision was still uncertain, the label would be determined by an experienced ROP expert (G.Z.). Finally, the images with agreeing labels were kept for the automated system training, validation, and test data sets. Images with disagreeing labels were excluded. In addition, the optic disc and blood vessels for preplus/plus disease identification were labeled by an experienced grader.

### Classifiers and Platform Development

The pipeline of our system is shown in eFigure 2 in the Supplement. An image was first evaluated for image quality. If it was predicted as ungradable, a recommendation to rephotograph was given. If the image was predicted as gradable, it entered the main pipeline. The main structure of the system was a multilabel classification^20^ and a postprocessing method that aggregated single-dimensional results to image-level results and image-level results to eye-level and patient-level results using max pooling. On a single image, ROP diagnosis could be viewed as a multilabel classification in that 1 image can present multiple features (stages of ROP, hemorrhage, and preplus/plus disease) simultaneously. This classification system was implemented using multiple independent classifiers based on the binary relevance. Multilabel classification was implemented using multiple independent classifiers instead of 1 classifier, because preplus/plus disease classification was implemented using an independent and complex pipeline. Taking the model ensemble into consideration, every classification task was implemented using a set of different neural networks. Dynamic data resampling and cost-sensitive learning were used simultaneously to resolve the class imbalance. Model ensemble and test time image augmentation were used to improve accuracy and make predictions robust to small perturbations.^21^ Label smoothing^22^ was used to calibrate the predicted probabilities. Because preplus/plus diseases have fewer positive samples than other classifiers and the presence of preplus/plus disease is attributed only to the blood vessels in the posterior pole, preplus/plus classification was considered to be a fine-grained classification and was implemented using an independent pipeline. An input image first needed to be judged on whether it was, in fact, a posterior image. The nonposterior image was regarded as non–preplus/plus. If the image belonged to a posterior image, the blood vessels were extracted using a patch-based DL technique called Res-UNet.^23,24^ Moreover, the optic disc was detected using a Mask-RCNN,^25^ and the posterior regions were calculated based on the optic disc. Afterward, the blood vessels in the posterior pole area were cropped and input into the final classifier. A set of neural networks was used to classify whether the image was preplus/plus disease or not. More details are available in eMethods 1 in the Supplement.

Finally, the image-level referral decision was automatically generated by integrating the results of multiple classifiers, and the eye-level and patient-level referral decisions were generated by integrating multiple image-level results. Details of the methods, especially that of algorithm development, are shown in eMethods 2 and 3 in the Supplement.

### Statistical Analysis

The data set was randomly split into training, validation, and test data sets with a ratio of 75:10:15 by a patient-based split policy in order to ensure all images of a patient were allocated into the same sub–data set of any classifier. The test set was also used to evaluate the performance of the automated referral decision. The performance of each classifier was evaluated by true negative (TN), false positive (FP), false negative (FN), true positive (TP), F1 score, sensitivity, and specificity. The receiver operating characteristic (ROC) analysis and area under curve (AUC) with 95% CIs were also calculated. Two-sided 95% CIs with the Delong method for AUC were calculated using the open-source package pROC, version 1.14.0 (Xavier Robin). Data were analyzed from July 15, 2019, to June 24, 2020.

The comparison was carried out between our platform, JSIEC Platform for Retinopathy of Prematurity (J-PROP), and 3 experienced ROP experts (W.G., D.G., and T.L.) from JSIEC on 200 retinal images extracted randomly from the test set. Three ROP-related features were identified, and a diagnosis of referral-warranted (RW) ROP was generated automatically by J-PROP via integration of the feature identification results or generated manually from the results of the ROP experts. A criterion-standard diagnosis originated from the ground truth labels. The Cohen unweighted κ was calculated and displayed in the interobserver heat map with a conventional scale where 0.2 or less was considered to be slight agreement, 0.21 to 0.40 was labeled as fair, 0.41 to 0.60 was labeled as moderate, 0.61 to 0.80 was labeled as strong, and 0.80 to 1.0 was considered to be near-complete agreement. The indexes of TN, FP, FN, TP, F1 score, sensitivity, and specificity were also calculated.

## Results

Of 55 490 retinal images, 3241 (5.8%) were discarded because they were nonfundus photos, fundus photos imaged by devices other than RetCam, not ROP but other ocular diseases such as congenital cataract and retinoblastoma, and images without agreed-upon labeling. A total of 52 249 retinal images from 14 108 eyes of 8652 preterm infants (mean [SD] gestational age, 32.9 [3.1] weeks; 4818 of 7973 boys with known sex [60.4%]) were annotated and included as the ground truth data set (Table 1). With the available data, the mean (SD) birth weight was 1925 (774) g. The data set was randomly split into training (n = 39 029), validation (n = 5140), and test (n = 8080) data sets with a ratio of 75:10:15 by a patient-based split policy. The demographic characteristics of the patients are shown in Table 1, and the data set distribution is listed in eTable 1 in the Supplement.

**Table 1.** Demographic Details of Patients^a^ From 4 Centers

Device	No./total No. (%)							
	Total	JSIEC			Yuexiu	Panyu	Qingyuan
	RetCam^b^ II & III	RetCam II & III	RetCam II	RetCam III	RetCam III	RetCam III	RetCam III
Patient, No.	8652	3714	2533	1181	2155	1251	1532
Images, No.	52 249	15 382	9225	6157	11 269	14 708	10 890
Eyes, No.	14 108	5907	3964	1943	3521	2225	2455
Patient sex known	7973/8652 (92.2)	3714/3714 (100)	2533/2533 (100)	1181/1181(100)	2058/2155 (95.5)	766/1251 (61.2)	1435/1532 (93.7)
Boys	4818/7973 (60.4)	2287/3714 (61.6)	1607/2533 (63.4)	680/1181 (57.6)	1295/2058 (62.9)	446/766 (58.2)	790/1435 (55.1)
Patients with GA available	6364/8652 (73.6)	3545/3714 (95.4)	2399/2533 (94.7)	1146/1181 (97.0)	2056/2155 (95.4)	763/1251 (61.0)	0
GA, mean (SD), wk	32.9 (3.05)	32.7 (2.60)	32.72 (2.54)	32.62 (2.71)	32.8 (3.40)	34.0 (3.67)	NA
Patients with BW available	6363/8652 (73.5)	3546/3714 (95.5)	2400/2533 (94.7)	1146/1181 (97.0)	2056/2155 (95.4)	761/1251 (60.8)	0
BW, mean (SD), g	1925 (774)	1885 (677)	1889 (742)	1879 (516)	1922 (931)	2118 (694)	NA

Abbreviations: BW, birth weight; GA, gestational age; JSIEC, Joint Shantou International Eye Center; NA, not available.

^a^ A total of 566 of 6364 infants (8.89%) were term infants with a GA of 37 weeks or older. The data of postmenstrual age was only available from 2457 infants of JSIEC, and the mean (SD) postmenstrual age was 40.6 (3.07) weeks. Images were taken from 1 visit of each infant randomly. The average (range) number of images per eye was 3 (1-11 images).

^b^ RetCam II and III are corneal contact retinal cameras used to photograph the retina. They are manufactured by Clarity Medical Systems.

### System Performance

**Figure 1.**
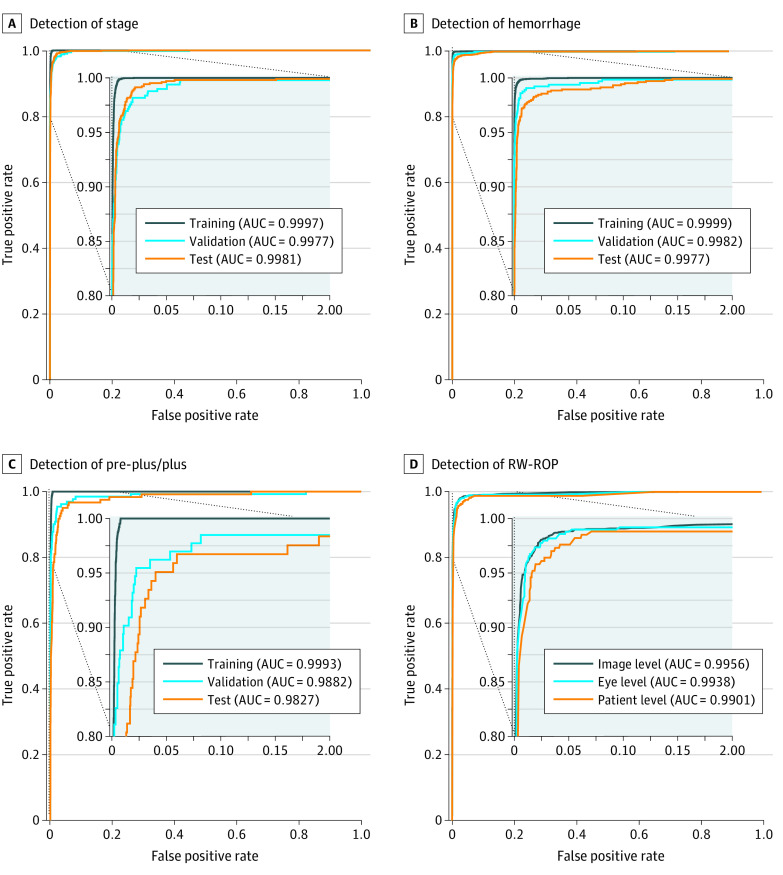
The Receive Operating Characteristic (ROC) Curves for System Performance The ROC for detecting any stage of retinopathy of prematurity (ROP) (A), intraocular hemorrhage (B), and preplus/plus disease (C). The area under curve (AUC) of training, validation, and test sets from each of the classifiers are shown (D). The ROC for referral-warranted ROP (RW ROP) was obtained by aggregating the results of 4 classifiers (stage, hemorrhage, posterior, and preplus/plus). Any positive findings of ROP-related features would result in the RW, and the AUC at the image, eye, and patient levels are shown.

The performance of 5 independent classifiers was validated and tested. In the test set, all classifiers achieved an F1 score of 0.718 to 0.981, a sensitivity of 0.918 to 0.982, a specificity of 0.949 to 0.992, and an AUC of 0.9827 to 0.9981 (Table 2, Figure 1, and eFigure 3 in the Supplement). For the ROP-related features, any stage of ROP achieved an F1 score of 0.946, a sensitivity of 0.982, a specificity of 0.985, and an AUC of 0.9981 (95% CI, 0.9974-0.9989), whereas hemorrhage achieved 0.961, 0.972, 0.992, and 0.9977 (95% CI, 0.9963-0.9991), respectively. The performance of preplus/plus disease achieved an F1 score of 0.718, a sensitivity of 0.918, a specificity of 0.970, and an AUC of 0.9827 (95% CI, 0.9706-0.9948).

**Table 2.** Performance of 5 Classifiers of the Platform

Classifiers	Data set	No.				F1	Sensitivity	Specificity	AUC (95% CI)
		TN	FP	FN	TP				
Image quality	Training	2771	23	849	35386	0.988	0.977	0.992	0.9976 (0.9973-0.9979)
	Validation	306	21	156	4657	0.981	0.968	0.936	0.9899 (0.9874-0.9924)
	Test	558	30	253	7239	0.981	0.966	0.949	0.9922 (0.9906-0.9938)
Stage	Training	31076	334	2	4823	0.966	1.000	0.989	0.9997 (0.9996-0.9998)
	Validation	4253	67	14	479	0.922	0.972	0.984	0.9977 (0.9959-0.9994)
	Test	6349	98	19	1026	0.946	0.982	0.985	0.9981 (0.9974-0.9989)
Hemorrhage	Training	30800	167	11	5257	0.983	0.998	0.995	0.9999 (0.9999-0.9999)
	Validation	4123	40	8	642	0.964	0.988	0.990	0.9982 (0.9957-1.0000)
	Test	6389	54	29	1020	0.961	0.972	0.992	0.9977 (0.9963-0.9991)
Posterior	Training	27203	861	488	7683	0.919	0.940	0.969	0.9936 (0.9931-0.9941)
	Validation	3558	153	70	1032	0.902	0.936	0.959	0.9908 (0.9890-0.9927)
	Test	5629	220	127	1516	0.897	0.923	0.962	0.9901 (0.9884-0.9918)
Preplus/plus	Training	12701	192	0	631	0.868	1.000	0.985	0.9993 (0.9991-0.9996)
	Validation	1707	27	12	120	0.860	0.909	0.984	0.9882 (0.9753-1.0000)
	Test	2518	78	10	112	0.718	0.918	0.970	0.9827 (0.9706-0.9948)
RW									
Images	Test	5295	144	40	2013	0.956	0.981	0.974	0.9956 (0.9942-0.9970)
Eyes		1694	83	7	482	0.915	0.986	0.953	0.9938 (0.9898-0.9977)
Patients		1047	68	6	327	0.898	0.982	0.939	0.9901 (0.9835-0.9966)
RW without hemorrhage^a^									
Images	Test	6193	156	28	1115	0.924	0.976	0.975	0.9958 (0.9943-0.9973)
Eyes		1902	75	4	285	0.878	0.986	0.962	0.9959 (0.9927-0.9991)
Patients		1183	61	3	201	0.863	0.985	0.951	0.9937 (0.9884-0.9991)

Abbreviations: AUC, area under curve; F1, F1 score; FN, false negative; FP, false positive; ROP, retinopathy of prematurity; RW, referral warranted; TN, true negative; TP, true positive.

^a^ Ignoring the hemorrhage dimension, 3 levels of RW ROP were regenerated based on the results of stage and preplus/plus classifiers in the test set.

In the test data set, the performance of RW ROP detection at the image level achieved an F1 score of 0.956, a sensitivity of 0.981, a specificity of 0.974, and an AUC of 0.9956 (95% CI, 0.9942-0.9970). Eye-level and patient-level F1 scores decreased to 0.915 and 0.898, respectively, whereas the outcomes of other indexes were similar to that of the image level (Table 2 and Figure 1). The performance of RW ROP detection ignoring the hemorrhage dimension was also analyzed (Table 2). In addition, the performance on 2 subsets, the RetCam II and RetCam III sets, were analyzed separately (eTable 2 and 3 in the Supplement). For comparing the performance between the single model and the ensemble model, the AUC of the models was calculated, and the performance of the ensemble model was more accurate than that of the single model (for identifying stage, the ensemble model achieved an AUC of 0.9981 [95% CI, 0.9974-0.9989] compared with that of 0.9968-0.9971 by a single model; for identifying hemorrhage, the ensemble model achieved an AUC of 0.9977 [95% CI, 0.9963-0.9991] compared with that of 0.9940-0.9969 by a single model; for identifying preplus/plus disease, the ensemble model achieved an AUC of 0.9827 [95% CI, 0.9706-0.9948] compared with that of 0.9712-0.9809 by a single model) (eTable 4 in the Supplement).

### Visualization and Explainability

**Figure 2.**
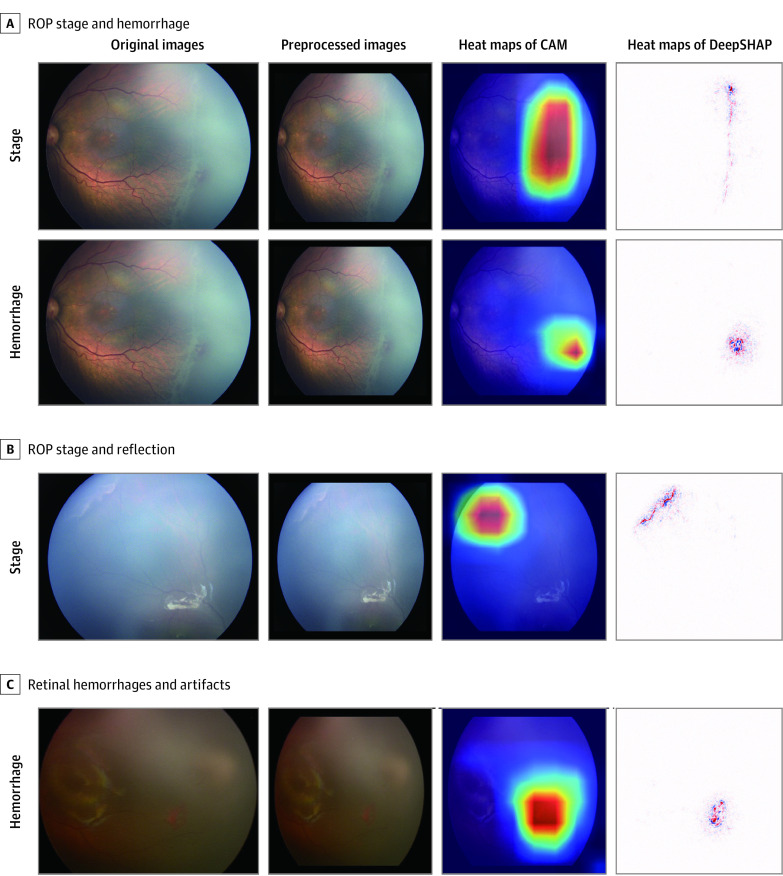
Visualization of Stage and Hemorrhage in Heat Maps The first and second columns indicate the original and preprocessed retinal images, respectively. The third and fourth columns are heat maps generated by Class Activation Mapping (CAM) and DeepSHAP, respectively. A, The original image presents both the stage of ROP and retinal hemorrhage on the peripheral retina. The upper row contains heat maps showing the stage of ROP, whereas the lower row contains heat maps showing retinal hemorrhages. B, The image presents both the stage of ROP and the reflection. Though it shares a similar morphology with its reflection, the lesion is successfully recognized. C, The image shows retinal hemorrhages and many artifacts; however, the hemorrhage area is highlighted by the heat map. DeepSHAP shows the more fine-grained heat map than CAM on each feature.

The features extracted by neural networks just before the classification header were visualized using t-Distributed Stochastic Neighbor Embedding, which is a technique for dimensionality reduction (eFigure 4 in the Supplement). DeepSHAP^31^ as a heat map technique was adopted to provide explainability, and extensive experiments were carried out to compare the heat maps generated by different techniques including DeepSHAP, Class Activation Mapping (CAM),^26,27^ Saliency Maps,^28^ Guided Backpropagation, Integrated Gradients,^29^ Layer-wise Relevance Propagation (LRP)-Epsilon, and LRP-Z^30^ (Figure 2 and eFigure 5 in the Supplement).

### Human-Platform Comparison

Our platform, J-PROP, was further compared with the ROP experts (Table 3). For the detection of intraocular hemorrhage, preplus/plus disease, and image-level RW ROP, J-PROP achieved a sensitivity of 1.000, whereas the experts achieved an average sensitivity of 0.958 to 1.000. The confusion matrix of the agreements among the ROP experts and criterion-standard diagnosis is shown in eFigure 6 in the Supplement. J-PROP achieved a Cohen κ of 0.93 for any stage of ROP, 0.97 for intraocular hemorrhage, 0.86 for preplus/plus disease, and 0.98 for RW ROP, whereas ROP experts achieved a mean Cohen κ of 0.93 (range, 0.87-1.00), 0.93 (range, 0.91-0.95), 0.98 (range, 0.95-1.00), and 0.95 (range, 0.93-0.99) for the 4 classifiers, respectively.

**Table 3.** Performance Comparison Between Human Experts and J-PROP

ROP-related features	Reader	No.				F1	Sensitivity	Specificity	AUC (95% CI)
		TN	FP	FN	TP				
Stage of ROP	Expert 1	152	0	0	48	1.000	1.000	1.000	NA
	Expert 2	152	0	9	39	0.897	0.812	1.000	NA
	Expert 3	148	4	3	45	0.928	0.938	0.974	NA
	Experts_average	NA	NA	NA	NA	0.943	0.917	0.991	NA
	J-PROP	148	4	1	47	0.949	0.979	0.974	0.9980 (0.9950-1.0000)
Intraocular hemorrhage	Expert1	156	4	1	39	0.940	0.975	0.975	NA
	Expert 2	160	0	3	37	0.961	0.925	1.000	NA
	Expert 3	155	5	1	39	0.929	0.975	0.969	NA
	Experts_average	NA	NA	NA	NA	0.943	0.958	0.981	NA
	J-PROP	158	2	0	40	0.976	1.000	0.988	1.0000 (1.0000-1.0000)
Preplus/plus disease	Expert 1	190	0	0	10	1.000	1.000	1.000	NA
	Expert 2	190	0	0	10	1.000	1.000	1.000	NA
	Expert 3	189	1	0	10	0.952	1.000	0.995	NA
	Experts_average	NA	NA	NA	NA	0.984	1.000	0.998	NA
	J-PROP	187	3	0	10	0.870	1.000	0.984	1.0000 (1.0000-1.0000)
RW ROP, image level	Expert 1	117	0	1	82	0.994	0.988	1.000	NA
	Expert 2	117	0	6	77	0.962	0.928	1.000	NA
	Expert 3	114	3	4	79	0.958	0.952	0.974	NA
	Experts_average	NA	NA	NA	NA	0.971	0.956	0.991	NA
	J-PROP	115	2	0	83	0.988	1.000	0.983	0.9999 (0.9996-1.0000)
RW ROP, image level, without hemorrhage^a^	Expert 1	147	0	0	53	1.000	1.000	1.000	NA
	Expert 2	147	0	5	48	0.950	0.906	1.000	NA
	Expert 3	147	0	3	50	0.971	0.943	1.000	NA
	Experts_average	NA	NA	NA	NA	0.974	0.950	1.000	NA
	J-PROP	144	3	0	53	0.972	1.000	0.980	1.0000 (1.0000-1.0000)

Abbreviations: AUC, area under curve; F1, F1 score; FN, false negative; FP, false positive; J-PROP, Joint Shantou International Eye Center Platform for Retinopathy of Prematurity; NA, not available; ROP, retinopathy of prematurity; RW, referral warranted; TN, true negative; TP, true positive.

^a^ The results of referral-warranted ROP here were generated by ignoring the hemorrhage dimension.

### Misclassification Analysis

In the test set, the images misclassified by any independent classifier were analyzed case by case for possible reasons. Poor contrast and artifacts were the most common reasons for misclassification in the stage and hemorrhage classifiers, whereas atypical vessel morphology was the main reason in preplus/plus disease with FN predictions. There were some errors due to incorrect annotations labeled by the junior ophthalmologists instead of by the J-PROP (eTable 5, eFigure 7, and eFigure 8 in the Supplement).

After full validation and testing, the neural network models were deployed to the production environment, and our cloud-based platform, J-PROP, was built and is openly accessible (eFigures 9 and 10 in the Supplement).

## Discussion

Results from this study suggest that (1) we developed a cloud-based DL platform integrating multidimensional classification and multilevel referral strategies that has the potential to meet clinical needs; (2) preplus/plus disease classification was implemented using an independent pipeline; and (3) DeepSHAP, which is a combination of DeepLIFT^32^ and Shapley values, could be adopted to generate fine-grained and class-discriminative heat maps in real time. Collectively, our automated ROP screening system, J-PROP, covering 4 main dimensions (image quality, any stages of ROP, intraocular hemorrhage, and preplus/plus diseases), was not only associated with high accuracy in both single-dimensional classification and image-, eye- and patient-level referral decisions but also generated fine-grained and class-discriminative heat maps for the explainability. The J-PROP platform is an open platform and appears to be promising for ROP screening.

From the point of view on a single image, ROP diagnosis can be viewed as a multilabel classification,^20^ wherein 1 image can belong to multiple classes simultaneously. This classification was implemented using multiple independent classifiers based on binary relevance (neglecting class dependence). With multiple images, ROP diagnosis is a multiple-instance learning problem^17^ wherein the images are instances and the eyes or patients are labeled as “bags.” Our study adopted a multimodal learning with decision-level fusion^33^ method, which differed from a previous study^16^ that used the standard multi-instance learning method. In the previous study’s method,^16^ the training instances come in bags, with all examples in a bag sharing the same label. A neural network has multiple inputs and a single output (Softmax was considered as a single output). Predictions were made only at the bag level. In contrast, a single-image classification with a postprocessing method was adopted in this study. The training instances were considered as singletons instead of bags. Predictions were made on the instance level, and the postprocessing method was used to generate the results of the bag level by aggregating (max pooling) the instance level results. The label of a single image can be given based on this image alone. As we labeled every image, J-PROP can fully use the samples, and neural networks can be trained quickly. On the contrary, for the traditional multiple-instance method, likely only 1 of multiple images was used to train the feature extractor during 1 backpropagation. In addition to the eye-level results, J-PROP has the potential to provide image-specific results. The explainable heat map of an image would not interfere with that of other images.

Preplus/plus disease classification is challenging and easily confused with the ROP stage or hemorrhage. For the preplus/plus disease classification, there are fewer positive samples than negative samples. Most of the preplus/plus disease images simultaneously contain the features of any stage of ROP or intraocular hemorrhages. Because preplus/plus disease classification is essentially related to the blood vessels in the posterior pole of the retina, preplus/plus disease classification was considered to be a fine-grained classification and was implemented using an independent pipeline, including blood vessel segmentation, optic disc detection, selection of the blood vessels of the posterior pole region, and preplus/plus disease classification. This design was based on domain knowledge, and the core idea aimed to allow the inputs of the preplus/plus disease classifier containing only the region of interest and removing the irrelevant features as much as possible.

The reasons for the false predictions on ROP-related features in the test set are shown in eTable 5 in the Supplement. Lesions with poor contrast and artifacts were the 2 common factors to interfere with the recognition of the staging of ROP and hemorrhage detection that led to FN and FP predictions. For preplus/plus disease, atypical morphology was the common cause of misclassification. Notably, various proportions of FP and FN were caused by incorrect annotations, which may have been due to the poor contrast, artifacts, and atypical morphologies as commonly found in DL platforms. Although J-PROP was not affected by artifacts in most of the cases, artifacts and atypical morphologies could still result in false predictions. In the future, we will work to continuously improve the generalization ability by adding more specific samples, such as images with different kinds of artifacts and with atypical morphologies. We hope to adopt a hard negative mining technique so as to pay more attention to the existing difficult samples.

### Limitations

There are several limitations in this study. First, according to the International Classification of Retinopathy of Prematurity, there are 5 stages of ROP (stage 1-5) and 3 levels of plus disease (normal, preplus, and plus). However, in this study, each class was divided into only 2 levels (ie, any stage or nonstage and preplus/plus or non–preplus/plus). Second, posterior pole zones I to III proposed by the International Classification of Retinopathy of Prematurity represent an important parameter affecting the severity of ROP. We did not include the zone factor in our classification. Third, in some cases, DeepSHAP heat maps are fragile, just as many other methods, and do not meet the sensitivity and implementation invariance^29^ at the same time. Fourth, our work did not include a consideration of cost and staff training to acquire the images. We focused on the development and validation of a cloud-based ROP platform. In the future, we hope to design an edge and cloud platform and to complete the protocol on running-cost use and imaging technician selection and training. Finally, in addition to the local explanations, global understanding of neural networks is needed. Future studies should focus on the following questions: what patterns learned by the neural networks could represent the stages of ROP, and how are the features extracted by the neural network matched to these patterns? Even though the global interpretability of neural networks is an open question, future studies should attempt to understand neural networks in detail by using global interpretability methods, such as activation maximization and filter visualization.

## Conclusions

This diagnostic study developed a cloud-based DL platform integrating a multidimension classification and multilevel referral strategy for ROP screening and referral recommendation. Results suggest that the referral decision could be automatically generated at the image, eye, and patient level. Our platform, J-PROP, has the potential to be applied in neonatal intensive care units, children’s hospitals, and rural primary health care centers for routine ROP screening. It may be useful in remote areas lacking in ROP expertise.

## Notes

Supplement. **eFigure 1.** Workflow of Retinal Images Annotation and Split Process **eFigure 2.** The Pipeline of Image-Based Automated ROP Screening System **eFigure 3.** The Receiver Operating Characteristic (ROC) Curves for System Performance **eFigure 4.** The T-Distributed Stochastic Neighbor Embedding (t-SNE) of 5 Classifiers **eFigure 5.** Visualization of 7 Mainstream Heat Maps **eFigure 6.** Interobserver Comparison Heat Maps **eFigure 7.** Representative Images and Reasons With False Negative Predictions Generated by Platform on 3 ROP-Related Features **eFigure 8.** Representative Images and Reasons With False Positive Predictions Generated by Platform on 3 ROP-Related Features **eFigure 9.** The Topology Structure of the Platform **eFigure 10.** Screenshots of Application Procedures on Cloud-Based ROP Screening Platform **eTable 1.** Dataset Distribution of 5 Dimensions **eTable 2.** Performance of 5 Classifiers Based on Image Set of RetCam II **eTable 3.** Performance of 5 Classifiers Based on Image Set of RetCam III **eTable 4.** The Performance Comparison of Each Classifier Between Single Model and Model Ensemble in the Test Set **eTable 5.** The Reasons of Misclassification on ROP-Related Features in the Test Set **eMethods 1.** Dataset Development **eMethods 2.** Deep Learning Algorithm Development **eMethods 3.** Deployment and Code/Data Availability **eReferences**
