# Supplementary material for: Automated Explainable Multidimensional Deep Learning Platform of Retinal Images for Retinopathy of Prematurity Screening
Source: JAMA Netw Open. 2021 May 5;4(5):e218758. doi: 10.1001/jamanetworkopen.2021.8758 (PMC8100867; doi:10.1001/jamanetworkopen.2021.8758)
Supplement: Supplement. — eFigure 1. Workflow of Retinal Images Annotation and Split Process eFigure 2. The Pipeline of Image-Based Automated ROP Screening System eFigure 3. The Receiver Operating Characteristic (ROC) Curves for System Performance eFigure 4. The T-Distributed Stochastic Neighbor Embedding (t-SNE) of 5 Classifiers eFigure 5. Visualization of 7 Mainstream Heat Maps eFigure 6. Interobserver Comparison Heat Maps eFigure 7. Representative Images and Reasons With False Negative Predictions Generated by Platform on 3 ROP-Related Features eFigure 8. Representative Images and Reasons With False Positive Predictions Generated by Platform on 3 ROP-Related Features eFigure 9. The Topology Structure of the Platform eFigure 10. Screenshots of Application Procedures on Cloud-Based ROP Screening Platform eTable 1. Dataset Distribution of 5 Dimensions eTable 2. Performance of 5 Classifiers Based on Image Set of RetCam II eTable 3. Performance of 5 Classifiers Based on Image Set of RetCam III eTable 4. The Performance Comparison of Each Classifier Between Single Model and Model Ensemble in the Test Set eTable 5. The Reasons of Misclassification on ROP-Related Features in the Test Set eMethods 1. Dataset Development eMethods 2. Deep Learning Algorithm Development eMethods 3. Deployment and Code/Data Availability eReferences [file jamanetwopen-e218758-s001.pdf]

Supplementary Online Content

Wang J, Ji J, Zhang M, et al. Automated explainable multidimensional deep learning platform of retinal images for retinopathy of prematurity screening. *JAMA Netw Open*. 2021;4(5):e218758.
doi:10.1001/jamanetworkopen.2021.8758

eFigure 1. Workflow of Retinal Images Annotation and Split Process
eFigure 2. The Pipeline of Image-Based Automated ROP Screening System
eFigure 3. The Receiver Operating Characteristic (ROC) Curves for System Performance
eFigure 4. The T-Distributed Stochastic Neighbor Embedding (t-SNE) of 5 Classifiers
eFigure 5. Visualization of 7 Mainstream Heat Maps
eFigure 6. Interobserver Comparison Heat Maps
eFigure 7. Representative Images and Reasons With False Negative Predictions Generated by Platform on 3 ROP-Related Features
eFigure 8. Representative Images and Reasons With False Positive Predictions Generated by Platform on 3 ROP-Related Features
eFigure 9. The Topology Structure of the Platform
eFigure 10. Screenshots of Application Procedures on Cloud-Based ROP Screening Platform
eTable 1. Dataset Distribution of 5 Dimensions
eTable 2. Performance of 5 Classifiers Based on Image Set of RetCam II
eTable 3. Performance of 5 Classifiers Based on Image Set of RetCam III
eTable 4. The Performance Comparison of Each Classifier Between Single Model and Model Ensemble in the Test Set
eTable 5. The Reasons of Misclassification on ROP-Related Features in the Test Set
eMethods 1. Dataset Development
eMethods 2. Deep Learning Algorithm Development
eMethods 3. Deployment and Code/Data Availability
eReferences

This supplementary material has been provided by the authors to give readers additional information about their work.

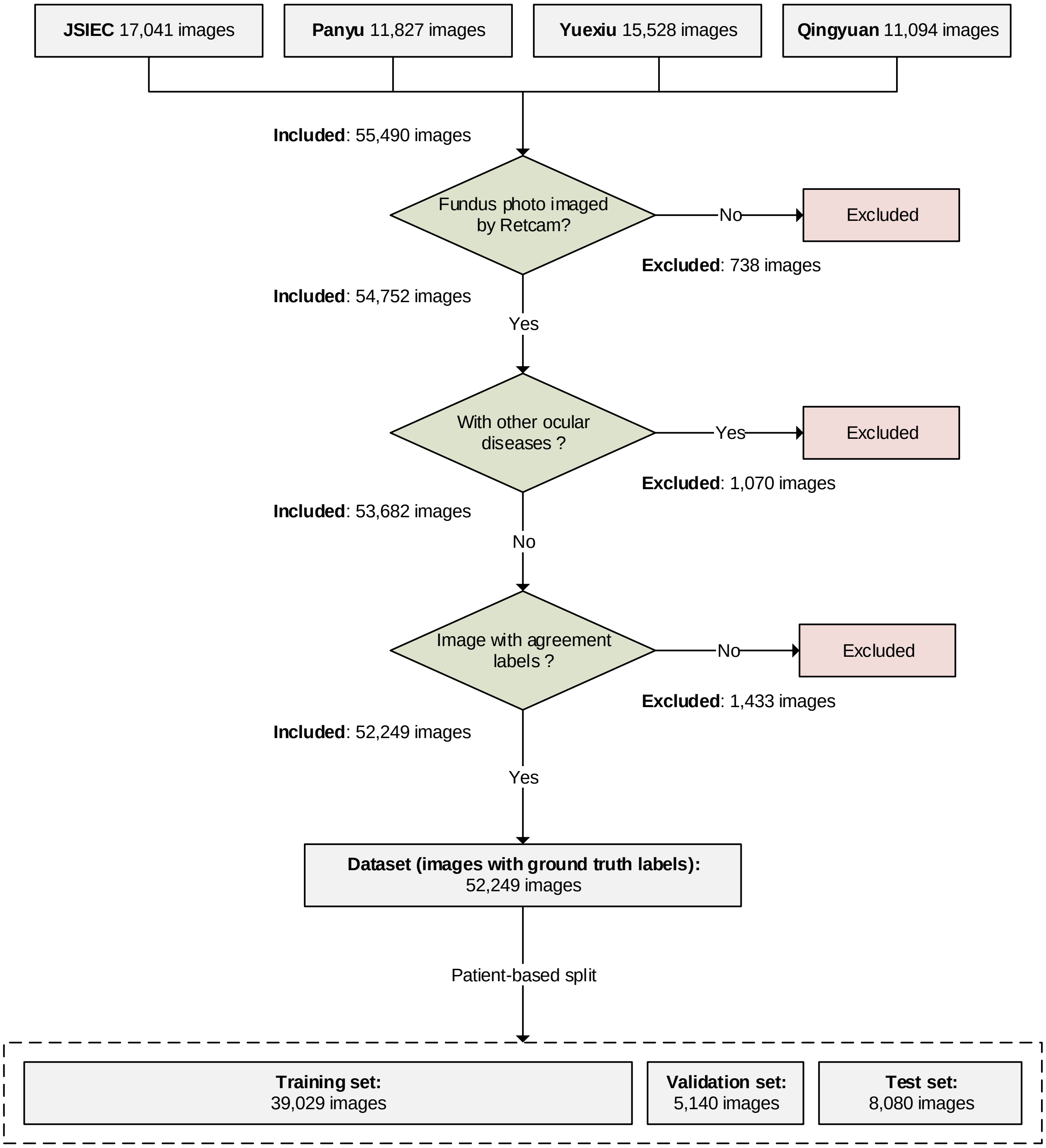

eFigure 1. Workflow of Retinal Images Annotation and Split Process
Initially, 55,490 retinal images were collected from four centers of China taken by RetCam (wide-field digital camera). Finally, total 52,249 images were included in training, validation and testing, after excluded some images as following: (1) non-fundus photos or fundus photo taken by imaging devices other than RetCam, (2) infants with other ocular diseases, including congenital cataract, retinoblastoma and persistent hyperplastic primary vitreous, and (3) any images with disagreement labels.

© 2021 Wang J et al. *JAMA Network Open.*

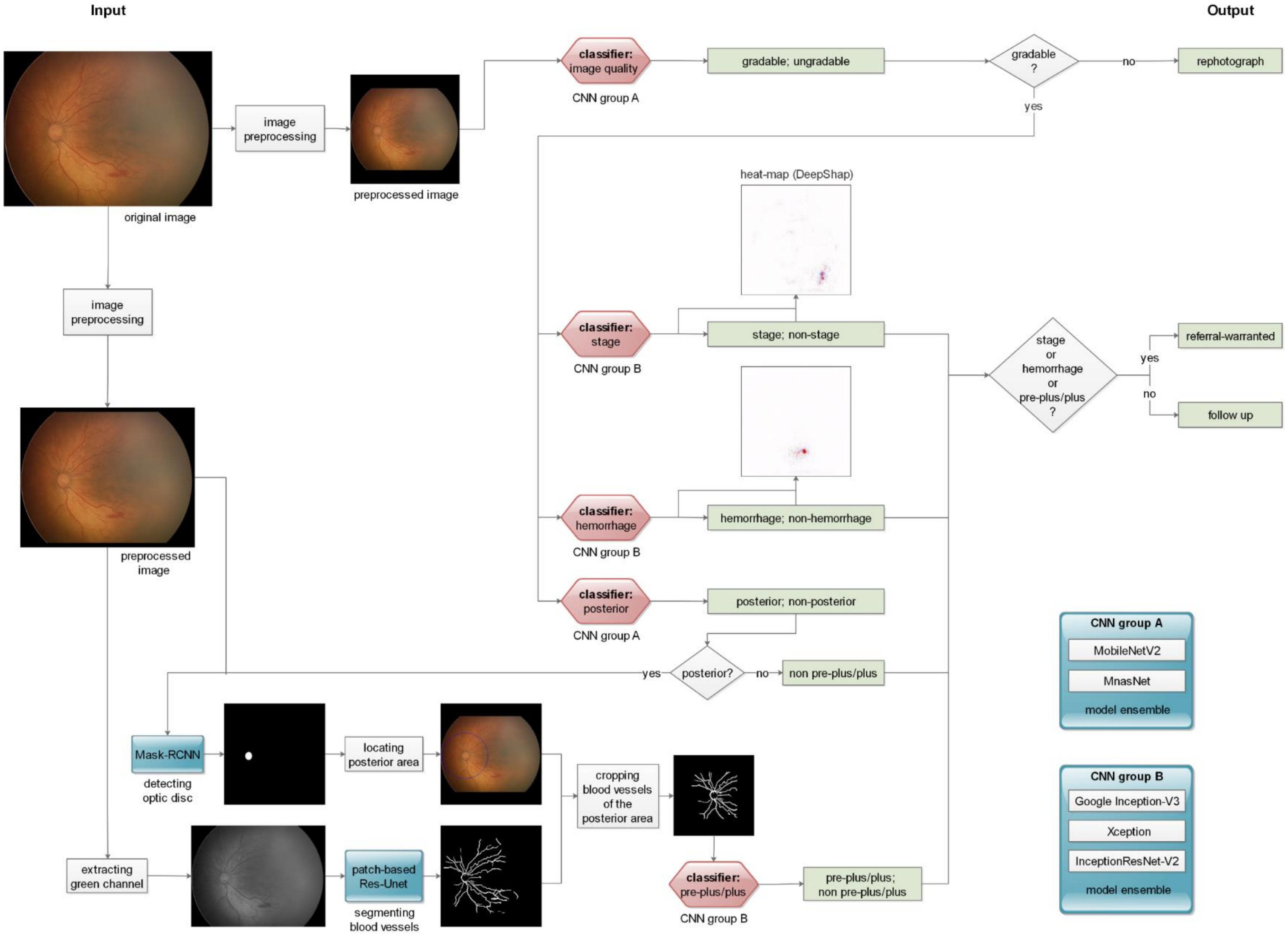

eFigure 2. The Pipeline of Image-Based Automated ROP Screening System

Given a retinal image, 5 dimensions would be evaluated using multiple independent classifiers. Firstly, image quality is evaluated, if quality probability value below the pre-defined threshold (such as 0.5), the image would be classified as “ungradable” and recommended for rephotographing. Otherwise, it is classified as “gradable” and will enter the main pipeline. Multiple independent classifiers based on the binary relevance were implemented on an image. When evaluating pre-plus/plus disease, an input image first needs to be judged whether it would be a posterior image. If yes, blood vessels and were extracted using a patch-based Res-Unet based on the green channel grayscale image extracted from the preprocessed color image. And then, the posterior region was detected using a Mask-RCNN. After that, posterior pole area blood vessels were cropped, and then a set of neural networks was used to classify the image as pre-plus/plus or non pre-plus/plus. The present or absence of three ROP-related features would be outputted, and any positive result of them would result in the “referral-warranted”, whereas all negative predictions results in the “follow-up”. Heat-maps would generate when there is any positive prediction for stage and hemorrhage.

© 2021 Wang J et al. *JAMA Network Open.*

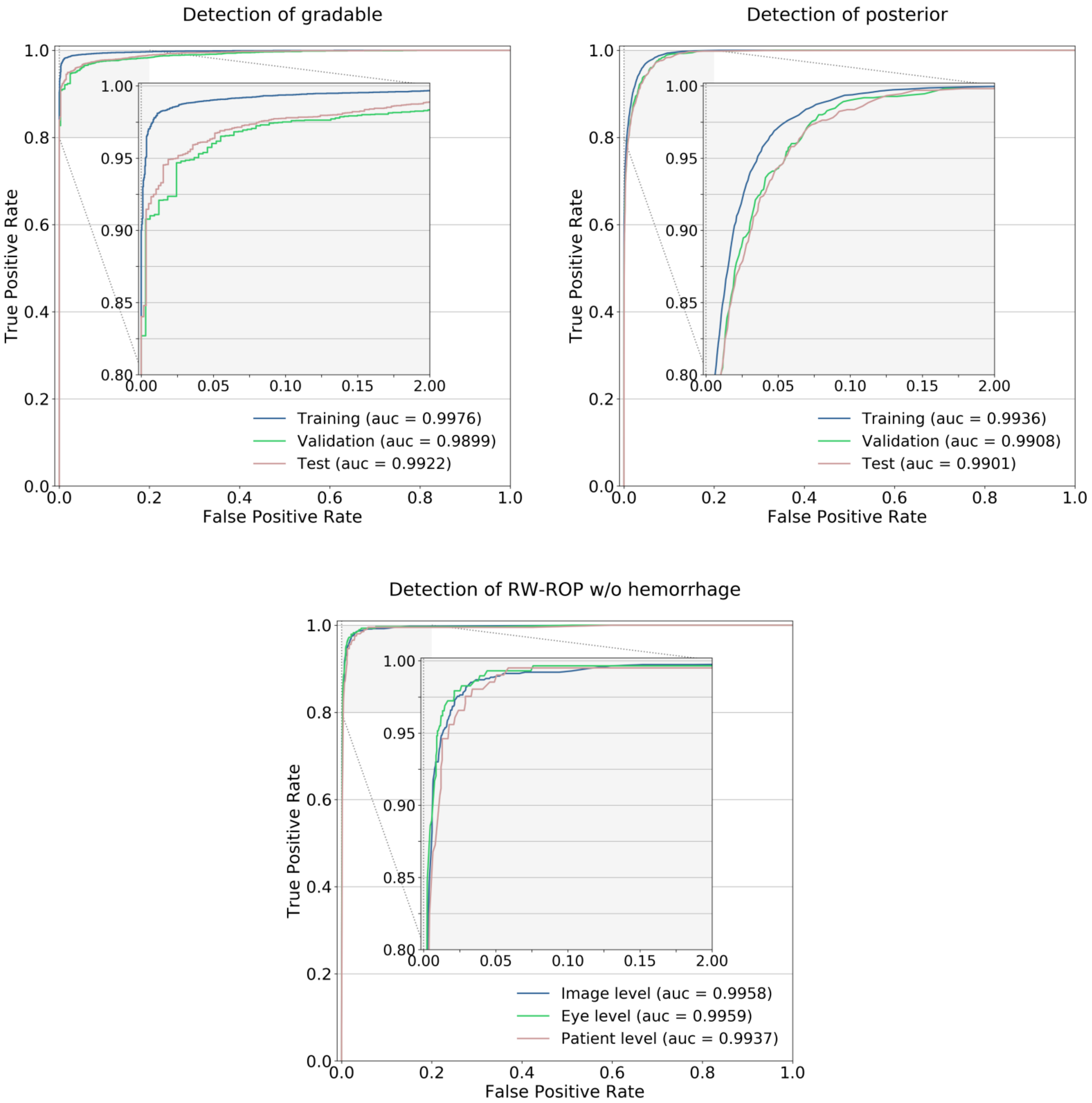

eFigure 3. The Receiver Operating Characteristic (ROC) Curves for System Performance

The ROC for detecting gradable image quality (upper left) and posterior pole area (upper right). The area under curve (AUC) of training, validation and test sets from each classifier are showed respectively too. The ROC for detecting referral-warranted ROP (RW) without hemorrhage dimension (lower) are showed in image-, eye- and patient-level , respectively. Only positive findings of stage and pre-plus/plus disease would result in the RW.

© 2021 Wang J et al. *JAMA Network Open.*

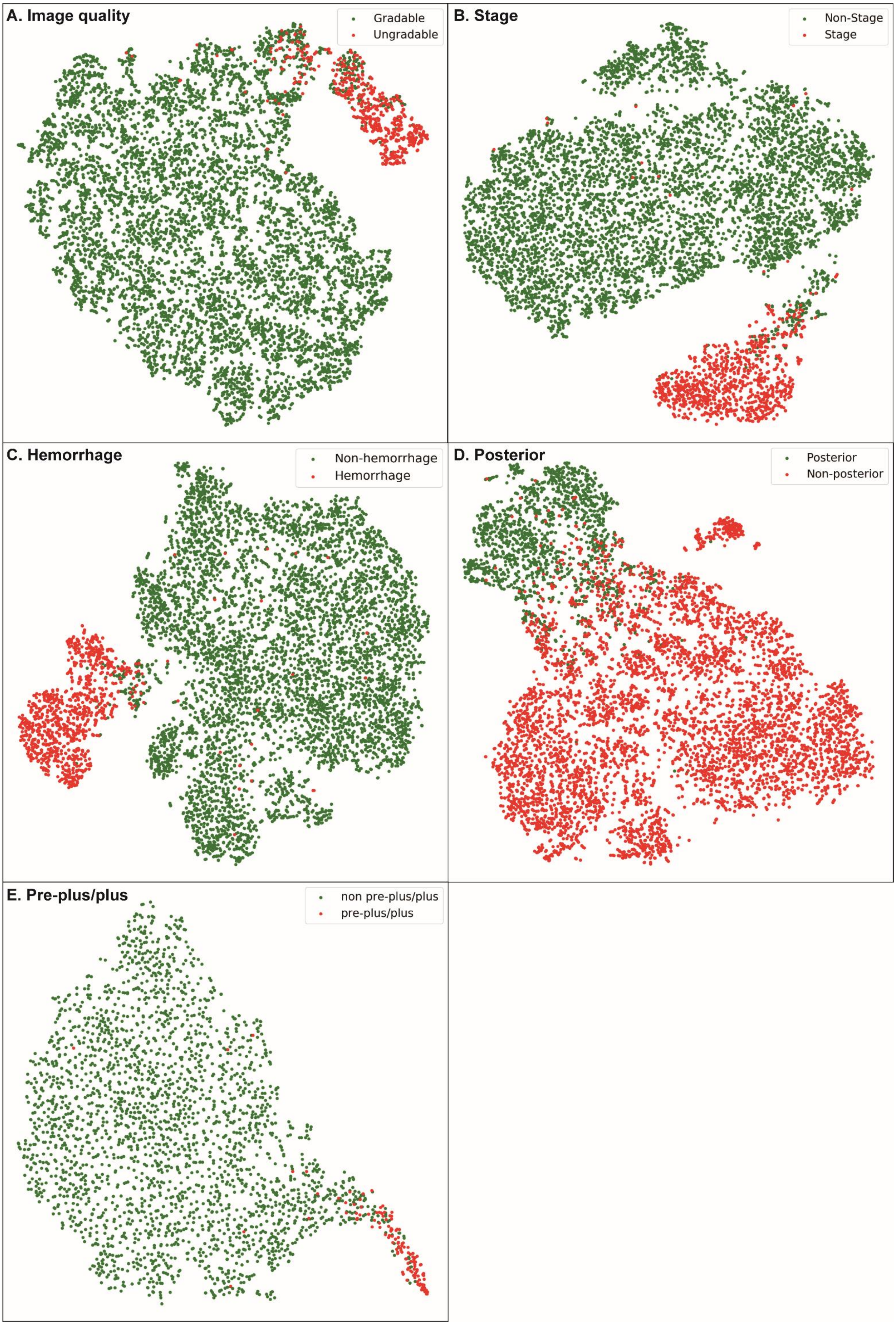

eFigure 4. The T-Distributed Stochastic Neighbor Embedding (t-SNE) of 5 Classifiers

t-SNE map provides visualized insight for features extraction of neural networks by converting data from high-dimension to two-dimension. Two classes of each independent classifier were colored by red or green in t-SNE map, showing well separation.

© 2021 Wang J et al. *JAMA Network Open.*

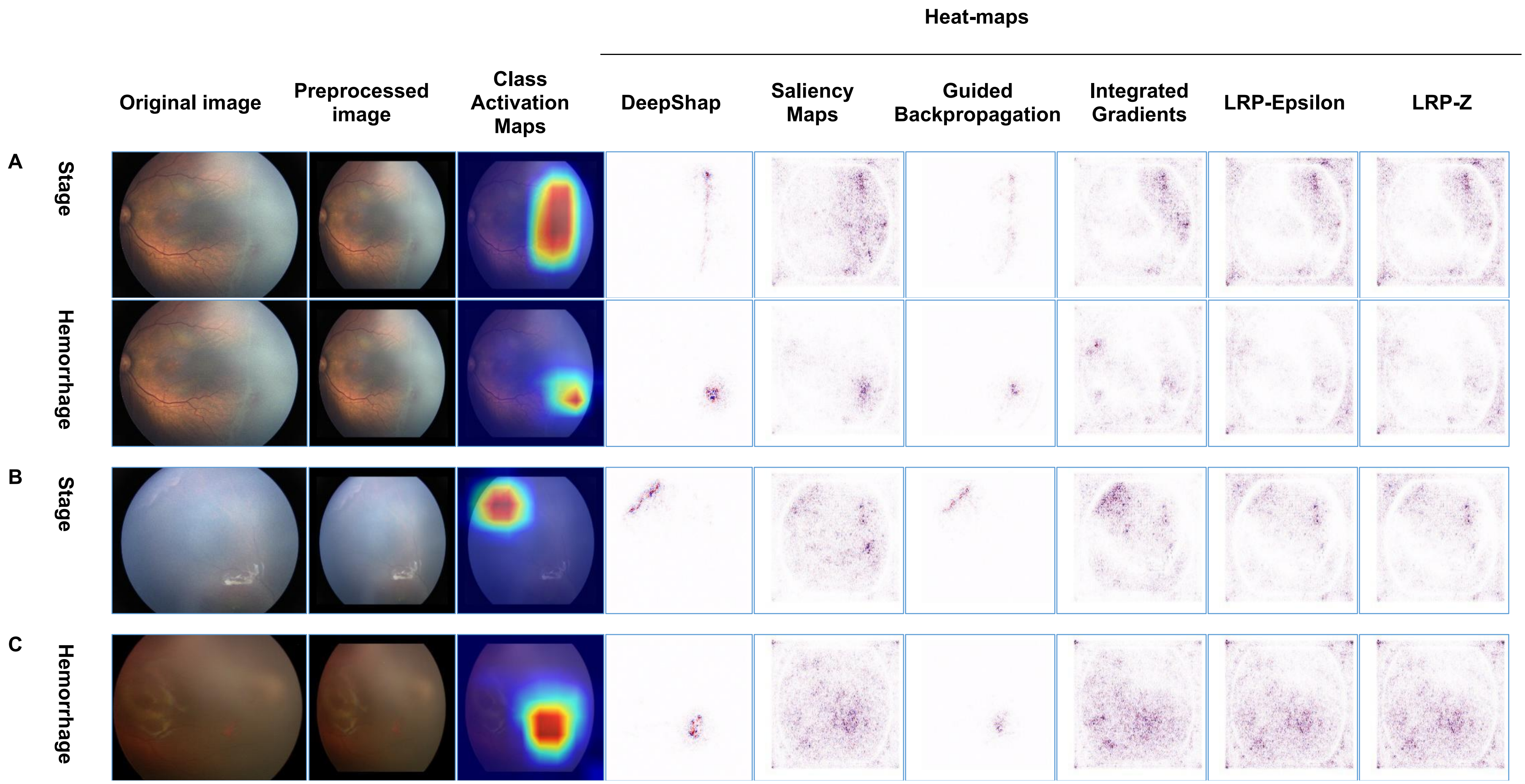

eFigure 5. Visualization of 7 Mainstream Heat Maps

Taking the same original images in Figure 2 as examples, 7 mainstream heat-maps technique for showing the area of stage and hemorrhage are listed from the 3rd to the 10th columns, respectively. Significantly, DeepShap generated the most fine-grained targeted area.

© 2021 Wang J et al. *JAMA Network Open.*

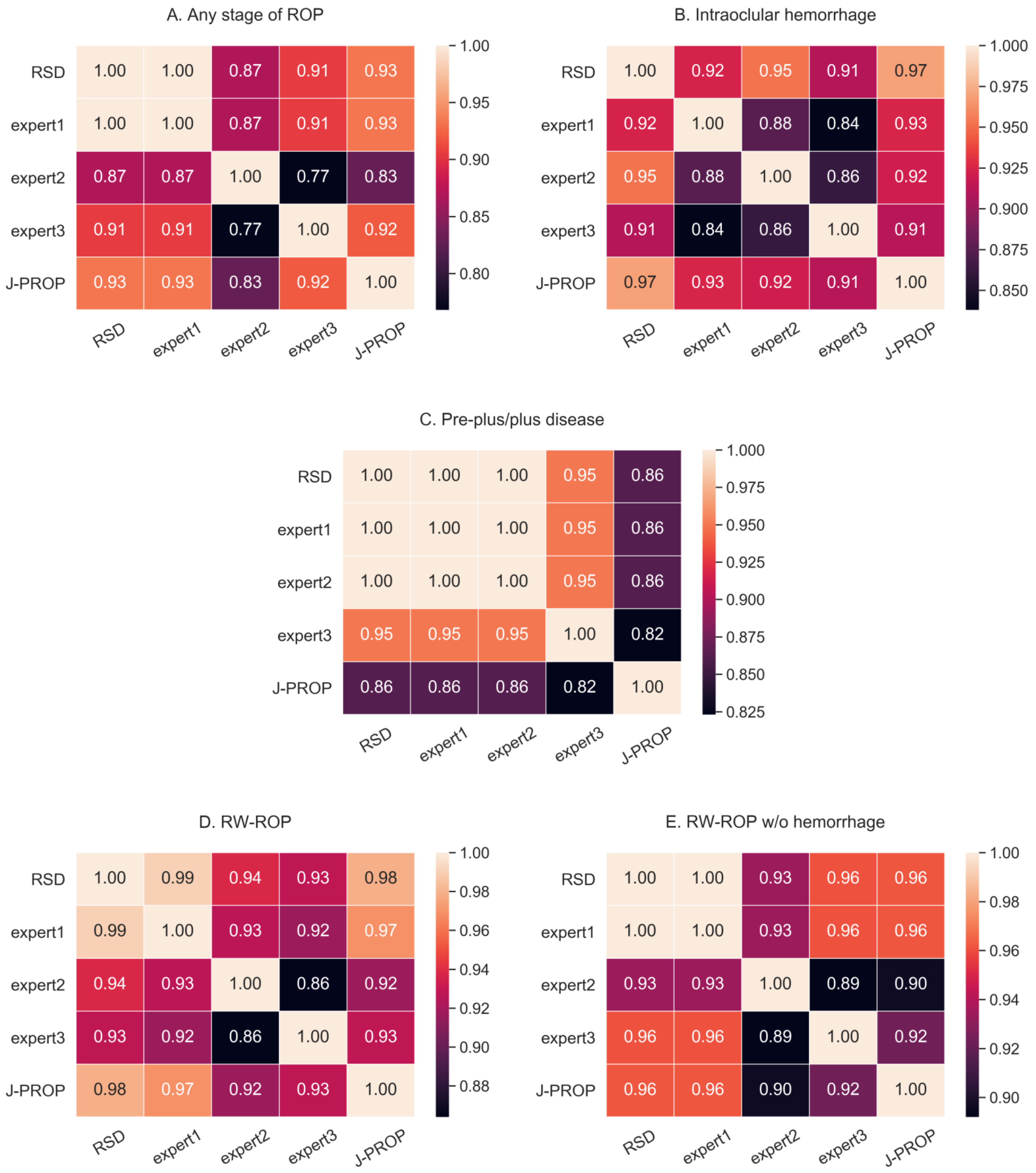

eFigure 6. Interobserver Comparison Heat Maps

The heat-maps showing kappa values of inter-observer agreement among 3 ROP experts, the platform (the Joint Shantou International Eye Center Platform for Retinopathy of Prematurity, J-PROP) and reference standard diagnosis (RSD) on three ROP-related features and referral-warranted ROP (RW-ROP) detection. Inter-observer heat-maps showing the performance of detection in any stage of ROP **(A)**, intraocular hemorrhage **(B)** and pre-plus/plus disease **(C)**, respectively. Inter-observer heat-map showing the detection in RW-ROP **(D)** after aggregating the results of three ROP-related feature, and the detection in RW-ROP ignoring hemorrhage-dimension **(E)**.

© 2021 Wang J et al. *JAMA Network Open.*

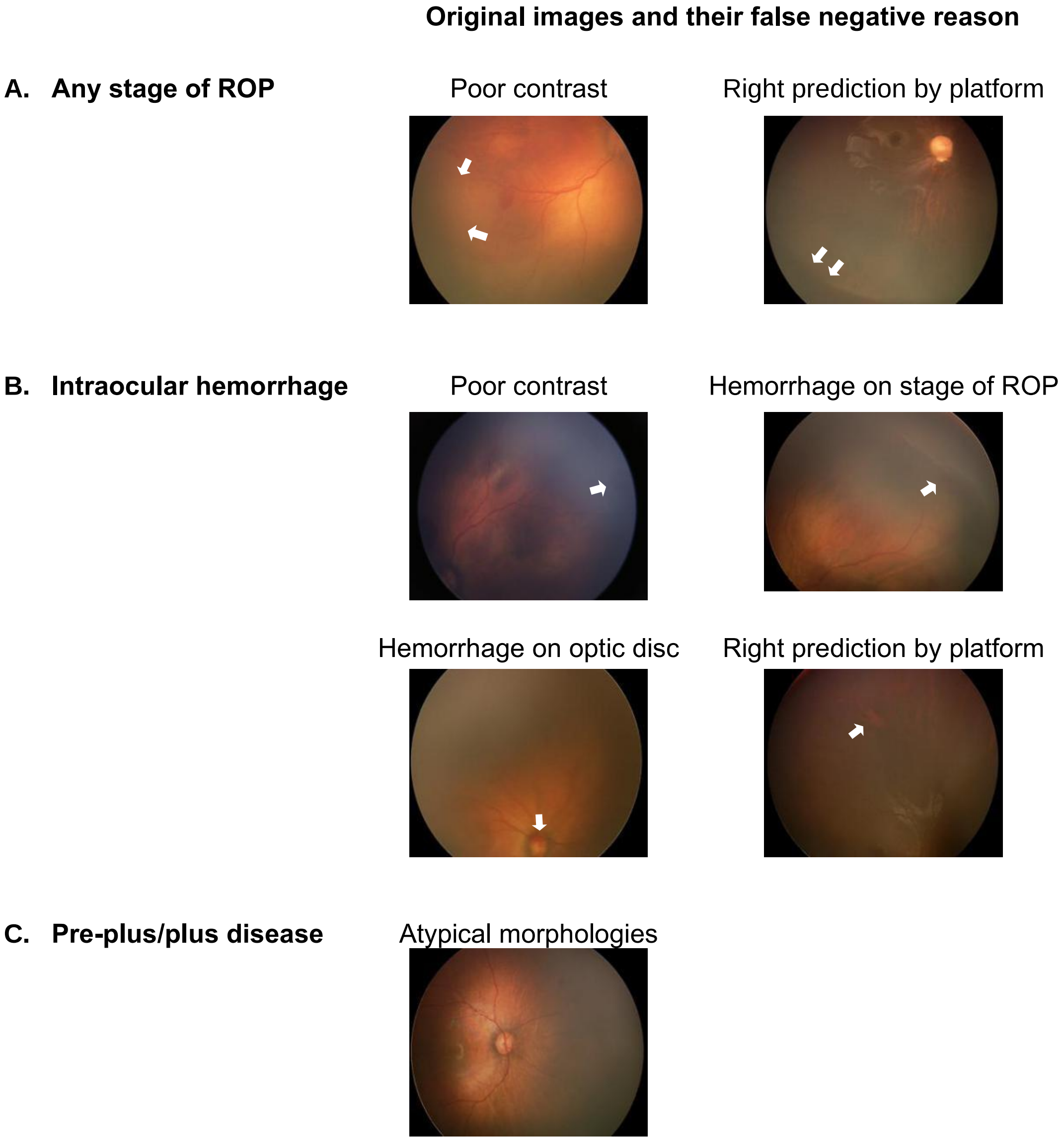

eFigure 7. Representative Images and Reasons With False Negative Predictions Generated by Platform on 3 ROP-Related Features

(A) For detecting any stage of ROP, total 19 false negative predictions were generated by platform. Lesion with poor contrast or similar to artifacts were the common reasons of misclassification, which account for 73.7% (n =14). 4 images with artifacts (21.1%) incorrectly annotated as stage-positive by human graders, however, were recognized rightly as normal by platform. **(B)** 29 images were of false negative predictions on hemorrhage by platform. Hemorrhages with the bad contrast were “neglected” by platform in 10 images (34.5%). Hemorrhage on stage-lesion (n =5, 17.2%) or optic disc (n =3, 10.3%), were misclassified as hemorrhage-free by platform. 10 images mis-labeled as hemorrhage-positive (34.5%) were rightly predicted as hemorrhage-free by platform, among which were 9 images with choroidal vessels (31.0%). **(C)** 10 images (100%) presenting atypical morphologies of posterior retinal vessels, due to physical variation, show mild dilated and tortuosity vessels which similar to pre-plus/plus disease. The atypical morphologies were mis-recognized as pre-plus/plus disease.

© 2021 Wang J et al. *JAMA Network Open.*

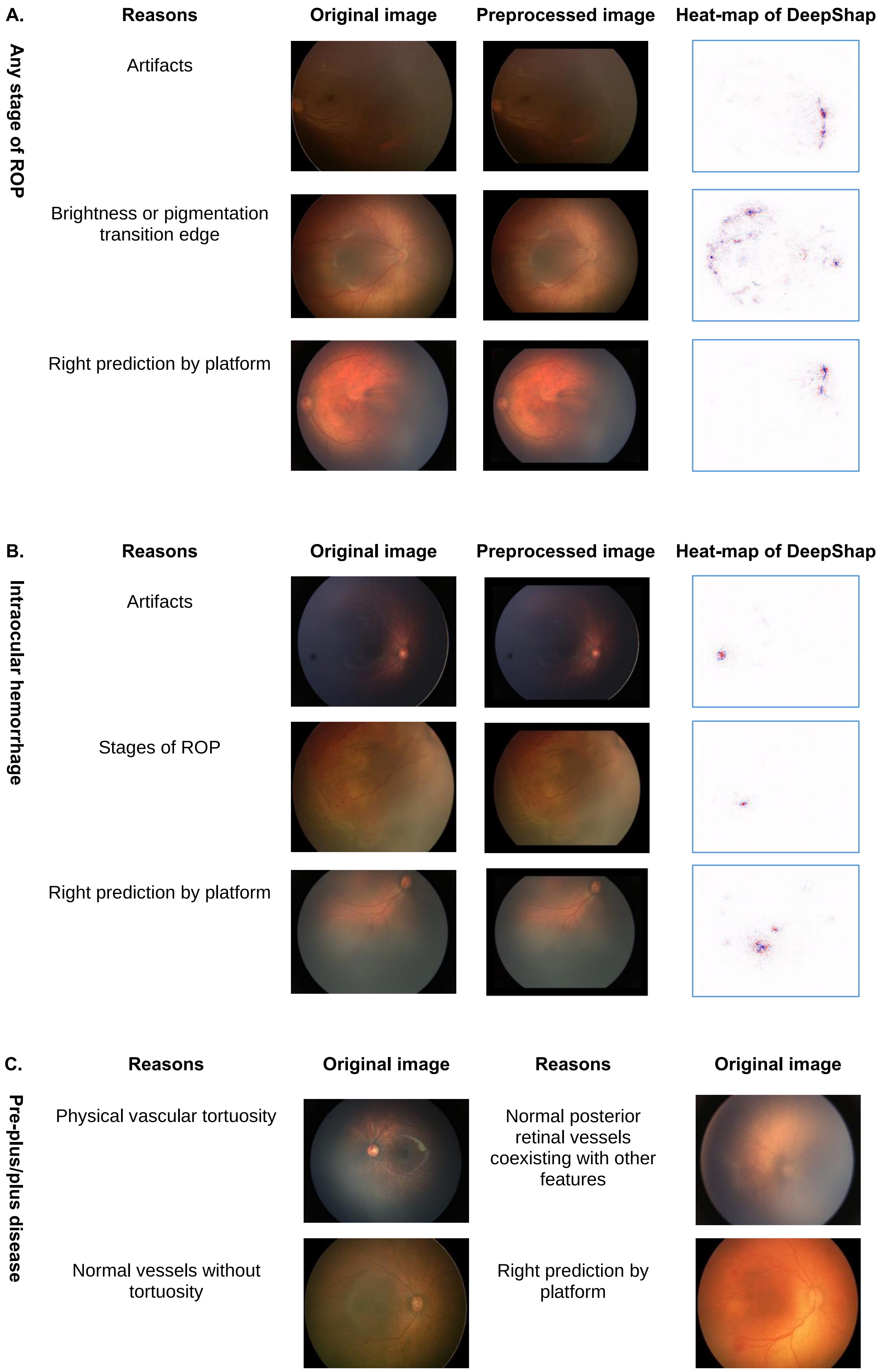

© 2021 Wang J et al. *JAMA Network Open.*

eFigure 8. Representative Images and Reasons With False Positive Predictions Generated by Platform on 3 ROP-Related Features

(A) Total 98 images were of false positive predictions by platform for detecting any stage of ROP. Artifacts (n = 46, 46.9%) and the transition edge of brightness or pigmentation (n = 29, 29.6%) were common reasons for resulting in false positive prediction by platform on stage-free images. Besides those, 21 images (21.4%) with wrong annotation by manual were predicted rightly by platform. **(B)** Artifacts (n = 10, 18.5%) were the major reason for false positive predictions on hemorrhage identification. 17 images (31.5%) with stage-lesion only also could result in false hemorrhage-positive predictions by platform. 23 images (42.6%) with hemorrhage, though annotated incorrectly by manual, were predicted rightly by platform. **(C)** Among 78 images with false positive prediction on pre-plus/plus disease, 32 (41.0%) were with physical vascular tortuosity, 22 (28.2%) with normal vessels without physiological variation, 22 (28.2%) with normal posterior retinal vessels coexisting other features, 2 (2.6%) with right prediction by platform.

© 2021 Wang J et al. *JAMA Network Open.*

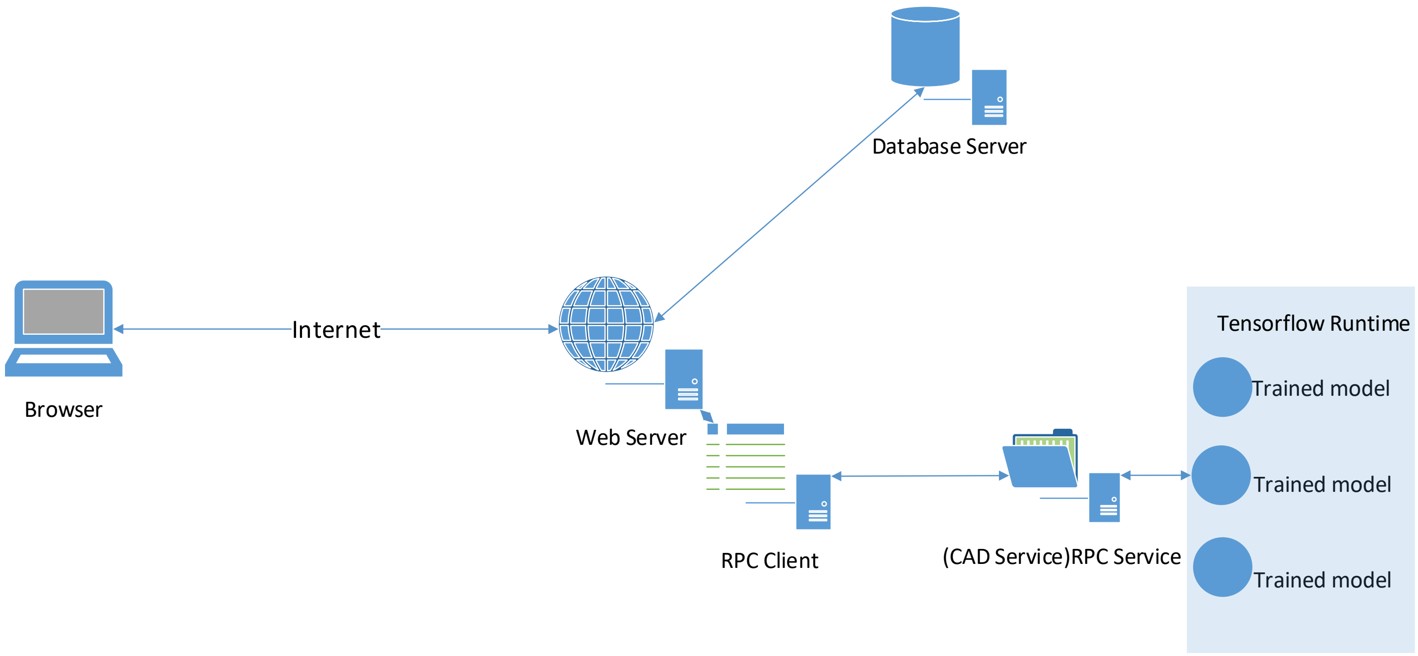

eFigure 9. The Topology Structure of the Platform
The platform contains Web application, Remote Procedure Call (RPC) Services and trained models.

© 2021 Wang J et al. *JAMA Network Open.*

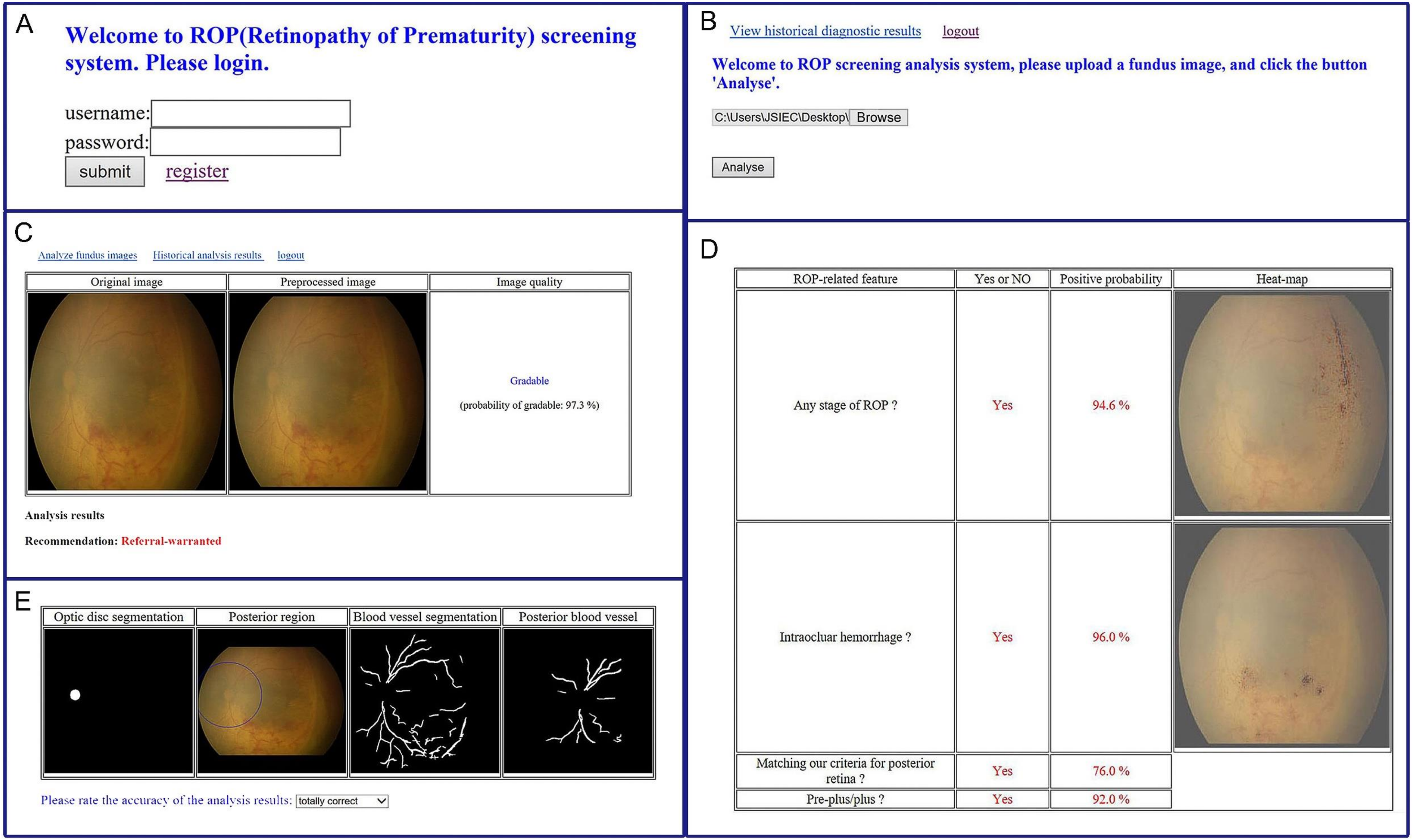

© 2021 Wang J et al. *JAMA Network Open*.

eFigure 10. Screenshots of Application Procedures on Cloud-Based ROP Screening Platform

(A) Login panel:

1. Please access the platform at http://113.106.224.28:8789, and input the pre-set login information as following:
   username: test
   password: jsiec
2. Click “submit” button.

(B) Upload page:

Please select and upload the image you want to analyse.

(C) Results page:

1. Firstly, image quality would be evaluated. Because the probability of 97.3% is more than the pre-defined threshold (such as 0.5), the image quality is gradable, which means the subsequent evaluation would be triggered.
2. Secondly, the recommendation following hybrid results is given below the original image and preprocessed image.

(D) Results page:

Thirdly, the predictions of each ROP-related feature were given along with a probability. Heat-maps would locate the lesion area when the predictive probability of classifiers of stage and hemorrhage exceed the threshold. In addition, pre-plus/plus would be evaluated subsequently if posterior area on image matches our criteria.

(E) Segmentation images of pre-plus/plus disease:

Finally, the segmentation images of optic disc, posterior region, blood vessel and posterior blood vessel segmentation would be given in turn, when evaluating pre-plus/plus disease.

© 2021 Wang J et al. *JAMA Network Open.*

eTable 1. Dataset Distribution of 5 Dimensions

Dimensions	Total, n	Labels (image, n)	Sets	n
Image quality	52,249	Gradable (n = 48,540)	Training	36,235
			Validation	4,813
			Test	7,492
		Ungradable (n = 3,709)	Training	2,794
			Validation	327
			Test	588
Any stage of ROP	48,540	Any stage (n = 6,363)	Training	4,825
			Validation	493
			Test	1,045
		Non-stage (n = 42,177)	Training	31,410
			Validation	4,320
			Test	6,447
Intraocular hemorrhage	48,540	Hemorrhage (n = 6,967)	Training	5,268
			Validation	650
			Test	1,049
		Non-hemorrhage (n = 41,573)	Training	30,967
			Validation	4,163
			Test	6,443
Posterior retina	48,540	Posterior (n = 10,916)	Training	8,171
			Validation	1,102
			Test	1,643
		Non-posterior (n = 37,624)	Training	28,064
			Validation	3,711
			Test	5,849
Pre-plus/plus disease	18,108	Pre-plus/plus (n = 885)	Training	631
			Validation	132
			Test	122
		Non pre-plus/plus (n = 17,223)	Training	12,893
			Validation	1,734
			Test	2,596

Total 52,249 retinal images were included and initially labeled based on image quality by manual. Only gradable quality images (n = 48,540) entered the subsequent dimensions labeling, including any stage of ROP, intraocular hemorrhage and posterior retina. Since pre-plus/plus disease was identified based on the posterior retinal vessel change, all images presenting standard posterior retina (n = 10,916) were employed for developing classifiers of pre-plus/plus disease. Moreover, some images without standard posterior retina presenting (n = 7,192) were randomly selected and added into the subset of pre-plus/plus disease for enhancing the generalization.

© 2021 Wang J et al. *JAMA Network Open.*

eTable 2. Performance of 5 Classifiers Based on Image Set of RetCam II

Classifiers	Dataset	NO.				F_1	Sensitivity	Specificity	AUC (95% CI)
		TN	FP	FN	TP				
Image quality	Training	429	7	160	6281	0.987	0.975	0.984	0.9967 (0.9956-0.9977)
	Validation	29	6	32	869	0.979	0.964	0.829	0.9828 (0.9742-0.9915)
	Test	54	8	70	1280	0.970	0.948	0.871	0.9787 (0.9699-0.9876)
Stage	Training	5894	46	0	501	0.956	1.000	0.992	0.9999 (0.9999-1.0000)
	Validation	798	14	2	87	0.916	0.978	0.983	0.9955 (0.9892-1.0000)
	Test	1236	6	8	100	0.935	0.926	0.995	0.9990 (0.9982-0.9998)
Hemorrhage	Training	6228	18	1	194	0.953	0.995	0.997	0.9998 (0.9996-1.0000)
	Validation	855	8	4	34	0.850	0.895	0.991	0.9947 (0.9894-1.0000)
	Test	1325	1	3	21	0.913	0.875	0.999	0.9969 (0.9920-1.0000)
Posterior	Training	3708	186	88	2459	0.947	0.965	0.952	0.9935 (0.9923-0.9947)
	Validation	517	38	15	331	0.926	0.957	0.932	0.9863 (0.9809-0.9917)
	Test	791	44	26	489	0.933	0.950	0.947	0.9913 (0.9883-0.9944)
Pre-plus/plus	Training	3618	66	0	342	0.912	1.000	0.982	0.9991 (0.9985-0.9996)
	Validation	502	9	5	89	0.927	0.947	0.982	0.9959 (0.9925-0.9993)
	Test	688	28	9	83	0.818	0.902	0.961	0.9771 (0.9598-0.9943)
RW, images	Test	1131	29	15	175	0.888	0.921	0.975	0.9879 (0.9803-0.9955)
RW, eyes		478	19	5	56	0.824	0.918	0.962	0.9812 (0.9624-1.0000)
RW, patients		309	17	4	41	0.796	0.911	0.948	0.9715 (0.9415-1.0000)
RW w/o hemorrhage, images[a]	Test	1134	28	17	171	0.884	0.910	0.976	0.9898 (0.9834-0.9961)
RW w/o hemorrhage, eyes[a]		481	18	4	55	0.833	0.932	0.964	0.9878 (0.9736-1.0000)
RW w/o hemorrhage, patients[a]		312	16	3	40	0.808	0.930	0.951	0.9820 (0.9592-1.0000)

Abbreviations: TN, true negative; FP, false positive; FN, false negative; TP, true positive; F1, F1 score; AUC, area under curve; C.I., confidence interval; RW, referral warranted; w/o, without.
[a] Ignoring the hemorrhage dimension, three levels of referral warranted ROP were re-generated based on the results of stage and pre-plus/plus classifiers in test set.

© 2021 Wang J et al. *JAMA Network Open.*

eTable 3. Performance of 5 Classifiers Based on Image Set of RetCam III

Classifiers	Dataset	NO.				F_1	Sensitivity	Specificity	AUC (95% CI)
		TN	FP	FN	TP				
Image quality	Training	2342	16	689	29105	0.988	0.977	0.993	0.9978 (0.9975-0.9981)
	Validation	277	15	124	3788	0.982	0.968	0.949	0.9907 (0.9881-0.9933)
	Test	504	22	183	5959	0.983	0.970	0.958	0.9936 (0.9921-0.9951)
Stage	Training	25182	288	2	4322	0.968	1.000	0.989	0.9996 (0.9995-0.9998)
	Validation	3455	53	12	392	0.923	0.970	0.985	0.9984 (0.9977-0.9992)
	Test	5113	92	11	926	0.947	0.988	0.982	0.9979 (0.9970-0.9988)
Hemorrhage	Training	24572	149	10	5063	0.985	0.998	0.994	0.9999 (0.9999-0.9999)
	Validation	3268	32	4	608	0.971	0.993	0.990	0.9984 (0.9957-1.0000)
	Test	5064	53	26	999	0.962	0.975	0.990	0.9975 (0.9959-0.9991)
Posterior	Training	23495	675	400	5224	0.907	0.929	0.972	0.9933 (0.9927-0.9939)
	Validation	3041	115	55	701	0.892	0.927	0.964	0.9915 (0.9895-0.9935)
	Test	4838	176	101	1027	0.881	0.910	0.965	0.9892 (0.9871-0.9913)
Pre-plus/plus	Training	9083	126	0	289	0.821	1.000	0.986	0.9995 (0.9993-0.9998)
	Validation	1205	18	7	31	0.713	0.816	0.985	0.9679 (0.9248-1.0000)
	Test	1830	50	1	29	0.532	0.967	0.973	0.9905 (0.9810-1.0000)
RW, images	Test	4164	115	25	1838	0.963	0.987	0.973	0.9964 (0.9951-0.9977)
RW, eyes		1216	64	2	426	0.928	0.995	0.950	0.9957 (0.9922-0.9993)
RW, patients		738	51	2	286	0.915	0.993	0.935	0.9932 (0.9873-0.9991)
RW w/o hemorrhage, images[a]	Test	5059	128	11	944	0.931	0.988	0.975	0.9969 (0.9956-0.9982)
RW w/o hemorrhage, eyes[a]		1421	57	0	230	0.890	1.000	0.961	0.9982 (0.9969-0.9994)
RW w/o hemorrhage, patients[a]		871	45	0	161	0.877	1.000	0.951	0.9972 (0.9951-0.9992)

Abbreviations: TN, true negative; FP, false positive; FN, false negative; TP, true positive; F1, F1 score; AUC, area under curve; C.I., confidence interval; RW, referral warranted; w/o, without.
[a] Ignoring the hemorrhage dimension, three levels of referral warranted ROP were re-generated based on the results of stage and pre-plus/plus classifiers in test set.

© 2021 Wang J et al. *JAMA Network Open.*

eTable 4. The Performance Comparison of Each Classifier Between Single Model and Model Ensemble in the Test Set

Classifier	AUC (95% C.I.)			
	**InceptionResnetV2**	**InceptionV3**	**Xception**	**InceptionResnetV2 & InceptionV3 & Xception**
Stage	0.9968 (0.9952-0.9984)	0.9971 (0.9957-0.9986)	0.9969 (0.9958-0.9980)	0.9981 (0.9974-0.9989)
Hemorrhage	0.9969 (0.9954-0.9983)	0.9940 (0.9904-0.9976)	0.9954 (0.9928-0.9980)	0.9977 (0.9963-0.9991)
Pre-plus/plus	0.9809 (0.9709-0.9910)	0.9724 (0.9528-0.9921)	0.9712 (0.9500-0.9925)	0.9827 (0.9706-0.9948)

Abbreviations: AUC, area under curve; C.I., confidence interval.

© 2021 Wang J et al. *JAMA Network Open.*

eTable 5. The Reasons of Misclassification on ROP-Related Features in the Test Set

**Any stage of ROP**	n	%
**False negative**		
Total	19	100.0
With poor contrast or similar to artifacts	14	73.7
Right prediction by platform	5	26.3
Artifacts	4	21.1
Pigmentations	1	5.3
**False positive**		
Total	98	100.0
Artifacts	46	46.9
Ora serrata	2	2.0
Brightness or pigmentation transition edge	29	29.6
Right prediction by platform	21	21.4
Poor contrast or similar to artifacts	21	21.4
**Intraocular hemorrhage**	n	%
**False negative**		
Total	29	100.0
With poor contrast	10	34.5
Hemorrhage on stage of ROP	5	17.2
Hemorrhage on macular	1	3.4
Hemorrhage on optic disc	3	10.3
Right prediction by platform	10	34.5
Artifacts	1	3.4
Choroidal vessels	9	31.0
**False positive**		
Total	54	100.0
Artifacts	10	18.5
Optic disc and pigmentation	4	7.4
Stages of ROP	17	31.5
Right prediction by platform	23	42.6
With poor contrast	23	42.6
**Pre-plus/plus disease**	n	%
**False negative**		
Total	10	100.0
Atypical morphologies of pre-plus/plus	10	100.0
**False positive**		
Total	78	100.0
Physical vascular tortuosity	32	41.0
Normal vessels without physiological variation	22	28.2
Normal posterior retinal vessels coexisting with other features	22	28.2
Right prediction by system	2	2.6
Atypical morphologies of pre-plus/plus	2	2.6

Some misclassifications were generated by three classifiers of ROP screening platform, including any stage of ROP, intraocular hemorrhage and pre-plus/plus disease. By visual inspection and/or heat-maps, the reasons of false negative and false positive of each classifier were summarized in the table.

© 2021 Wang J et al. *JAMA Network Open.*

eMethods 1. Dataset Development

Datasets

Retinal images of infants taken by RetCam II or III (Clarity Medical Systems, Pleasanton, CA) for ROP screening were collected from four centers in southern China: Joint Shantou International Eye Center of Shantou University and the Chinese University of Hong Kong (JSIEC) between 4th November 2010 -14th November 2019, Guangdong Women and Children Hospital in Yuexiu (Yuexiu) between 2nd January 2017 - 29th December 2018, Guangdong Women and Children Hospital in Panyu (Panyu) between 27th August 2018 - 4th January 2019 and the Sixth Affiliated Hospital of Guangzhou Medical University and Qingyuan People's Hospital (Qingyuan) between 20th November 2014 - 24th September 2019. Images were taken from one visit of each infant randomly. The average (range) number of images per eye was 3 (1-11). Compare to the normal images, the images presenting lesions are more valuable for image-based diagnosis. Besides, class imbalanced ratio should be controlled. Hence, based on the EMRs, 1-3 retinal images were randomly selected from each normal patient, whereas all retinal images were exported from the infants with ROP.

Intraocular hemorrhage

The specific lesions for retinopathy of prematurity (ROP) diagnosis have been defined in the revised version of the International Classification of Retinopathy of Prematurity (ICROP) in 2005, including stage 1-5 and pre-plus/plus disease. However, many challenges still hinder us to diagnose ROP based on retinal images:

1)The ROP lesions commonly lie on the peripheral retina, where was frequently imaged poorly to be identified, especially for the early stage of ROP.

2)Although the pre-plus/plus disease, locating at the posterior pole of retina, could be imaged under well exposure, it is hard to identify early with confidence due to the qualitative description by ICROP.

3)The media haze could be generated physiologically and pathologically, which could interfere the retinal imaging and confuse the judgment.[1]

On the contrary, intraocular hemorrhage, a non-specific lesion for ROP diagnosis, demonstrates multiple advantages for ROP detection based on images as follows:

1) Intraocular hemorrhage observed in the fundus, vitreous or on the retina of premature infants, implies the direct correlation with the presence and severity of ROP and poor prognosis.[2, 3]

2) Intraocular hemorrhage is easy to identify with consistency, which was proved by the perfect agreement of inter-observer from our human-platform comparison (Table 3);

3) A dense hemorrhage could still be identified through the media haze, whereas the specific lesions could be covered by media haze.

Collectively, intraocular hemorrhage as an aid for ROP detection could alert the image reader for interpreting the outcome cautiously to prevent under-diagnosis of ROP.

Posterior pole

ICROP shows a standard circle area with 3 diameters of optic disc for identify the pre-plus/plus disease. However, the report from the i-ROP research consortium suggested that, when centering the optic disc (OD), the widest field of view (6 disc diameter as radius) prompt the highest diagnosis accuracy on plus disease, when compare to narrower field (1-5 disc diameter as radius).[4] Besides, in Mao J et al. study, the circle area with 3 OD as radius was cropped to quantify parameters from posterior vessel for pre-plus/plus detection.[5]

The posterior pole defined in our study was the same as that in Mao J et al study. The reasons were as following:

1) Too narrow view as ICROP described decreases the sufficient information for pre-plus/plus disease detection;

2) Too wide view of posterior area may increase redundant and unreasonable information to confusing the diagnosis, which had been shown in human experts' comparison. [6]

3) In addition, the more width of view, the less image number can meet the demand.

In conclusion, the posterior pole is limited to the circle area with three OD as radius and the center of OD as center.

Subset of pre-plus/plus disease

According to the system workflow, only the posterior images were subjected to the classification of pre-plus/plus disease. For the non-posterior images, they were classified as "non pre-plus/plus" (or neglected the pre-plus/plus dimension). Hence, the sample number of pre-plus/plus dataset should be the same as that of posterior images. However, posterior classification was before the pre-plus/plus classification based on blood vessels in the posterior region. The dividing line between a posterior image and a non-posterior image is not absolute. From the point of view on the system robustness, the pre-plus/plus classifier should have the ability not to be disturbed by the non-posterior images, otherwise, if a non-posterior image was misclassified, the image may be further misclassified as pre-plus/plus (false positive). Consequently, beside the posterior images, some non-posterior images (n =7,192) randomly selected from the posterior dataset were added into the pre-plus/plus dataset as negative samples (eTable 1in Supplement).

eMethods 2. Deep Learning Algorithm Development

1. Image preprocessing

The image resolutions used for image classification and blood vessel segmentation were 299 x 299 and 640 x 512 pixels respectively. The algorithm of image preprocessing for image classification was shown as follows: the black background areas were cropped using a threshold method, followed by converting the image into square by adding black paddings. To avoid deleting meaningful areas during the image augmentation process, some black areas (5% of the side length of the image square) were added to the borders of the fundus images. The algorithm of image preprocessing used in blood vessel segmentation was slightly different from that used in classification. For the blood vessel segmentation, the original image was resized to 640 x 480, and converted to 640 x 512 by adding black paddings. Critically, the color images were converted to the grayscale images by extracting the green channel since the blood vessels were more prominent in the green channel.

© 2021 Wang J et al. *JAMA Network Open.*

2. Image classification

Even though there are only two classes in each ROP dimension, multi-class classification, instead of binary classification, was used for all classifiers since more classes (such as stage 1-5 of ROP) could be added in the future. Softmax was used as the last layer's activation function, and weighted categorical cross-entropy as the loss function.

2.1 Class imbalance

The imbalance ratios for image quality, stages of ROP, hemorrhage and pre-plus/plus disease datasets were 12.98, 6.28, 5.33 and 19.3 respectively. During training, the data level method (dynamic data re-sampling) and cost-sensitive method (weighted cross entropy loss function) were simultaneously used to resolve class imbalance.

2.2 Real time data augmentation

Real time data augmentation was used during both training and inference. In general, image augmentation was used more often during training than during test time. However, test time image augmentation has been used in ImageNet[7] (multi-crop) and Kaggle Data Science Bowl 2017 competitions[8]. It improved not only the accuracy but also the robustness to small image perturbations. As a patch-based method was used in blood vessel segmentation, test time image augmentation could improve the accuracy of patch border areas. Compared with beforehand image augmentation, real time image augmentation was flexible and simplified the whole training process. During training models except for that of the pre-plus/plus disease classification, both geometry transformations and lightness and color transformations were used in image augmentation. Specifically, the images were randomly rotated (range: [-15°, 15°]), translated (range:[-10%,10%]), scaled (range: [95%,105%]), horizontally and vertically flipped, and image contrast were modified (multiplicative factor range:[90%,110%]).During the training pre-plus/plus models, only geometry transformations were used because the input images are binary images. During inference, for an image, two other images were generated on the fly using pre-defined transformations. Specifically, one image was generated by moving (dx=6px, dy=6px) and horizontal flipping, and the other image was generated using moving (dx=-6px, dy=-6px) and vertical flipping. Training time image augmentation was implemented using the imgaug[9] library. However, test time image augmentation was implemented by custom designed OpenCV codes.

2.3 Dynamic data re-sampling

Compared with the traditional under-sampling, it makes full use of training data because in every epoch it generated a different training dataset on the fly. Compared with the traditional over-sampling, which simply copied samples, it could avoid over-fitting. Since dynamic re-sampling and real time augmentation were used together, for a minority class image, different images were generated on the fly using real time augmentation.

Dynamic data re-sampling algorithm:
Let S be the original training set. Let epoch_num be the epoch of training. Let num_classes be the number of classes of S.
Pseudo codes:

```
num_samples ←len(S)
FOR epoch_current=0 to epoch_num – 1
    Ŝ ← new set()   #Ŝ is the generated training set of the current epoch
    list_weights← [1, 2.5] # a list of class sampling weights, one element for every class.
    list_p←list_weights/sum(list_weights) #normalize the weights j← 0 #the number of samples already sampled
    WHILE j <num_samples
        k ←0#the index of the current sample in the original training dataset
        class1←the label of S[k]
        randomly select S[k] using probability list_p[class1]
        IF S[k] is selected
            Add S[k] to Ŝ
            j←j + 1
        ENDIF
        k←k + 1
        IF k == num_samples
            k← 0
        ENDIF
    ENDFOR
    yield Ŝ
ENDFOR
```

2.4 Convolutional neural networks

Two convolutional neural networks (CNN) groups, each of them contained the models of different architectures, were used for ROP image classification. Group A included MobileNetV2[10] and MnasNet,[11] and was used for image quality classification and posterior classification, which were relatively easy tasks. Group B included Inception-V3, [12] Xception[13] and InceptionResNet-V2[14], and were used for other tasks, including hemorrhage classification, stage of ROP classification and pre-plus/plus disease classification.

The design principle based on the standard models, instead of the custom designed models, can transfer from the ImageNet pre-trained models. To ensure the diversity of models, CNNs with different architectures were used in every CNN group. Ensemble learning was best suited for the models that are high accurate and different. [15]

As for CNNs in Group A, which do relatively easy classification tasks, the performance differences between different models were very small. MobileNetV2 and MnasNet were chosen as base models because they are lightweight and these two models are very different. By using lightweight models, memory consumption and FLOPS were reduced, and the training and prediction time were shortened. MobileNetV2 was designed by human, however MnasNet was invented by neural architecture search. We hope that using models with different architectures can reduce models correlations, so as to improve the performance of model ensemble (mostly by reducing variance).

As for CNNs in Group B, which were used to do main classification tasks, Inception-V3, Xception and InceptionResNet-V2 were chosen because of the following reasons.

© 2021 Wang J et al. *JAMA Network Open.*

The first two models were parameter and computation efficient. The last model combines the two most important architectures used in computer vision, i.e., Inception and Resnet, and achieved very high performance in a lot of tasks. All three models include the Inception architecture. For medical images, the size of the lesions varied greatly. The inception modules consist of multi-scale filtering units, and some researches hypothesize that inception models are good at extracting multi-scale features (although there may not be enough evidence about this). Depthwise separable convolutions proposed by Xception were widely used in a lot of neural networks including Mobilenet-V2. So far Inception-V3 is the most popular model used in fundus images analysis, and InceptionResNet-V2 is also widely used in fundus images analysis.

In the process of another project we did prior to this study, which utilized over 20,000 fundus images. A lot of models including Inception-V3, Xception, InceptionResNet-V2, Resnet50, NASNet, SE-Net and our custom designed model based on dilated convolution and attention mechanism were tried and found out that these three models were the best models in this scenario. The comparisons of the three models in Group B are as follows: From the point of view of statistical metrics, Inception-Resnet V2 obtained the best performance among the three models. It can't be ignored that the number of parameters and FLOPS needed by it were much more than those of the others two models. Inception-v3 and Xception gained similar performance metrics, however, heat-maps generated by Xception were better than that generated by Inception-v3.

2.5 Loss function

Weighted categorical cross-entropy was used as the loss function in order to tackle class imbalance and find a balance between false negatives and false positives. The formula of cross-entropy loss function was L = $-\sum_i \text{class\_weight}_i \cdot y_i \cdot \ln\bar{y}_i$. In this formula y_i was the ground truth probability for class i, $\bar{y}_i$ was the predicted probability for class i, and class_weight_i the weight of class i. Because of label smoothing, $y_i$was not always s$\epsilon\{0,1\}$ =. The class_weight = {0:1.0, 1:3.5}, which means weight 1 for negative class (class no. 0) and weight 3.5 for positive class (class no. 1) was used in the loss function of stage and hemorrhage classifications. Likewise the class_weight = {0:1.0, 1:4.5} was used in the loss function of pre-plus/plus disease classification.

2.6 Training

Transfer learning[16] was applied for training all models. The feature extractor of a model was initialized using the corresponding pre-trained ImageNet model, and all layers were then fine-tuned. Adam[17] with lookhead[18] (k = 5, alpha = 0.5) was used as the optimizer, with a custom learning rate scheduler to adjust learning rate dynamically. Label smoothing (ε=0.1) was used to calibrate probabilities[19].

2.7 Prediction Process

For an image, after being preprocessed, test time image augmentation and model ensemble were used to generate the final predicted probabilities. The mathematical formula is:

$$\text{probs\_final} = \frac{\sum_{i=1}^{n} \sum_{j=1}^{m} (W_i \times P_{ij})}{(\sum_{i=1}^{n} W_i) \times m}$$

The number of CNN models involved was denoted by n, and the number of test time image augmentation was by m. Wi was the weight of the model No. i. For simplicity, instead of being learned by a meta-learner[20], Wi was set as the square of the validation accuracy of model i. Pij was the predicted probability of model i for image augmentation j. Both parameter n and m were set to 3. Setting n and m to be greater than 3 would not bring obvious performance improvement. However it would result in consuming more computing power and long response time. The final predicted probability array was denoted by probs_final. Threshold moving was not adopted. The formula to generate class label from predicted probabilities: pred_class = probs_final.argmax (axis = -1).

This prediction method has both pros and cons. Pros: Compared to single model one single input image, it can improve performance. The prediction mechanism of deep neural networks is very different from that of human. Although deep neural networks have achieved very good performance results, they are fragile. Model ensemble and test time image augmentation can make predictions more robust to small perturbations than single model. It needs to be pointed out that this method can not defend generative adversarial attacks. Cons: Increase system complexity and require more computational resources.

2.8 t-SNE

A neural network can be described as a feature extractor with a classifier header. The features extracted by neural networks could be visualized using t-SNE. Because different neural networks generate features with different dimensions, model ensemble could not be applied in t-SNE. A 1-D feature vector (2048 for Xception) was extracted for every image, and a 2-D feature vector (num_samples, 2048) was generated for the whole dataset. After doing dimensionality reduction using t-SNE, high dimensional data were converted to low dimensional data (num_samples, 2), and then projected into a scatter plotimage. The Sklearn.manifold.t-SNE library was used to process the data, and the matplotlib library was used to generate the scatter plot images.

2.9 Explainability

The explainability of neural networks was critical, especially for the medical applications. DeepShap (DeepExplainer) a combination of Deeplift and Shapley value, was used to generate high-quality heat-maps. We compared the results from DeepShap[21] with those of other 6 mainstream heat-maps techniques, including, CAM, [22, 23] DeepShap, Saliency Maps, [24] Guided Backpropagation, Integrated Gradients, [25] LRP-Epsilon and LRP-Z. [26] Compared with CAM, it could generate fine-grained heat-maps. It was more efficient and generated better results than other approximation methods such as Layer-wise relevance propagation (LRP) and Integrated Gradients. Different from the original Deeplift and Integrated Gradients, a distribution of background samples, instead of a single reference value, was used in DeepExplainer. The number of references used was 24. Since it cannot fit into the memory of a GTX2080TI, a splitting and concatenating strategy was used to reduce memory usage per time. An animation file was created by blending the preprocessed image with the DeepExplainer heat-map, and the frame per second parameter was set to 1.

An image showing positive results in both ROP stage and hemorrhage was analyzed by 7 different heat-map techniques (eFigure 5 in the Supplement). The lesion-specific region shown by CAM was presented in red-yellow color with rough location. In contrast, DeepShap presented the lesion regions in red-blue color, which was almost identical to the shape of the lesion. For other 5 techniques, the performance of Guided Backpropagation was closely resembled to that of DeepShap; yet, Guided Backpropagation showed more noises and less fine-grained. Therefore, we selected DeepShap to generate a more fine-grained and class discriminative heat-map in our platform.

3. Blood Vessel Segmentation

Some technical methods used in blood vessel segmentation, such as image augmentation and model ensemble, were identical to that

© 2021 Wang J et al. *JAMA Network Open.*

used in image classification. We focused on the differences. Retinal blood vessel segmentation for adults and for preterm infants could be viewed as the same task but with different data distributions. Specifically, the image quality and vascular morphology of preterm infants were largely different from that of adults. A representation-based domain adaption was performed by a two-step transfer learning method. The training details were illustrated in the training section.

3.1 Patch-based Method

In order to enlarge the training dataset, a patch-based method was adopted. During training, for a 640 x 512 image, 180 patches (64 x 64) were generated. 80 of 180 patches were non-overlapping and extracted using a grid style clipping, and the remaining 100 patches were randomly clipped patches. During inference, only non-overlapping patches were used.

3.2 Semantic Segmentation Models

A modified Residual U-Net was used as the blood vessel segmentation model. Compared with the original U-Net, both the depth and width of the network were reduced. A down-sampling block in the encoder and an up-sampling block in the decoder were eliminated. Filters were reduced from [64, 128, 256, 512] to [32, 64, 128, 256]. The design principles were based on the following three facts: a small input image size 64 x 64 was used. Compared with other tasks, the blood vessel segmentation does not need a large field of view. Using small models not only speed up training and inference time, but also avoid over-fitting in some cases. Other minor modifications included using pre-activation ResNet structure and adding a batch normalization layer after every convolutional layer.

3.3 Loss function

A hybrid loss[27] consisting of a pixel-wise cross-entropy loss and a soft dice-coefficient loss[28] was used for as the loss function. The hybrid loss may have advantages of: smooth gradients and handling class imbalance. Mathematically, the hybrid loss was defined as:

$$\mathcal{L}_{total} = \mathcal{L}_{CE} + \alpha \times \mathcal{L}_{dice}$$

α is a balance factor between cross entropy and dice loss, and was empirically set to 3.

$$\mathcal{L}_{CE} = -(\mathrm{p}\log(\tilde{p}) + (1-\mathrm{p})\log(1-\tilde{p}))$$

$$\mathcal{L}_{dice} = 1 - \frac{2\sum_{pixels} p * \tilde{p}}{\sum_{pixels} p + \sum_{pixels} \tilde{p}}$$

In order to quantify |A| and |B|, the simple sum was used for this calculation instead of the squared sum.

3.4 Training

A two-step transfer learning method was used to implement the domain adaption. First, the models were trained using four publicly available blood vessel segmentation datasets, DRIVE, STARE, CHASE_DB1 and HRF. Secondly, the trained models in the first step were using the ROP blood vessel segmentation dataset. Adam with lookhead (k = 5, alpha = 0.5) was used as the optimizer, and a custom learning rate scheduler was used to adjust learning rate dynamically.

3.5 Prediction Process

The test time image augmentation and model ensemble methods were the same as that of image classification. Subsequently, the probability map, having the same size as the input image, was obtained. Afterwards, the probability map was converted into a binary image based on a pre-defined threshold (such as 0.5). Finally, a simple post-processing method, which deleted small disconnected areas, was used to refine the final image.

4. Optic disc segmentation and posterior area detection

4.1 Optic disc segmentation. Multiple previous studies have worked on adult optic disc and optic cup segmentation. At present, there is still no optic disc segmentation research on infants' fundus images. An instance-based domain adaption method was used to train ROP optic disc segmentation models. The optic disc segmentation dataset was formed by combining four datasets for adults and one dataset for ROP. A Mask R-CNN was used to detect and segment the optic disc. Instance segmentation was used instead of object detection and semantic segmentation since there was a large number of pixel-level annotated training samples and the confidence value of the detected optic disc was important.

4.2 Cropping posterior area.

The bounding box of a detected optic disc was denoted by (x1, y1, x2, y2), the center and diameter of the optic disc were calculated by:

circle_center = ((x1+x2)//2, (y1+y2)//2)

circle_diameter = (abs(x2-x1) + abs(y2-y1)) // 2

The posterior area was determined by drawing a circle using the circle center as the center and the 3.0 * circle_diameter as the radius. As soon as the posterior circle was determined, the posterior area image can be cropped from the original image.

5. Plus classification

Plus classification is a much more difficult task than any other classification tasks. In the research process of this study, we used a variety of methods and carried out extensive experiments. In the beginning, for plus classification, we use the same method as stage classification. In the condition of excluding positive samples of hemorrhage and stage, good results have been obtained even though these exists some kinds of over-fitting. However, after adding positive samples of hemorrhage and stage, the performance degraded greatly. A lot of hemorrhage or stage images were misclassified as plus. Later, we improved the algorithm, i.e, the pre-plus/plus disease classification was considered as a fine-grained classification and implemented using an independent pipeline, and achieved good results.

This method has two drawbacks:

1) It increases the complexity of the system so as to increase the response time of the system and the workload of system maintenance.

2) The final results of plus classification depend not only on the classifiers themselves but also on the predecessor tasks including blood vessel segmentation and optic disc localization, so as to greatly increase the complexity of system debugging. Optic disc localization is a relatively easy task, however, premature infant blood vessel segmentation is much more difficult than adult blood vessel segmentation because the image quality is much worse. Even though we have adopted a variety of technologies including modern neural network models, complex training method such as transfer learning and domain adaption, the performance of blood

vessel segmentation is not very good. Fortunately, plus classification doesn't need fine blood vessel segmentation, it only need to detect the features of blood vessel distortion and dilation

eMethods 3. Deployment and Code/Data Availability

Deployment

After training and validation, all CNN models were deployed for production. The simplified architecture of the cloud-based platform was shown in Figure 1. A custom designed computer-aided diagnosis service (CADS) was developed instead of using standard Tensorflow Serving because generating heat-maps needed low-level controls on models. The trained CNN models were automatically loaded during the start-up of the CADS. CADS provided services through the xmlrpc. Web server application was developed using Flask, and implemented an xmlrpc client which can communicate with CADS.

Code/data availability

The related algorithm codes are available at https://github.com/linchundan88/ROP. The deployed platform can be openly accessed at http://113.106.224.28:8789/ (username: test, password: jsiec).129 samples of an optic disc segmentation dataset based on premature infant retinal images are publicly available at https://data.mendeley.com/datasets/prcy36j53v. 100 images of a blood vessel segmentation dataset based on premature infant retinal images are publicly available at http://data.mendeley.com/datasets/tv3b2bt6dy.

eReferences

1. Ludwig CA, Greven MA, Moshfeghi DM. Predictors of treatment-warranted retinopathy of prematurity in the SUNDROP cohort: influence of photographic features. *Graefe's Archive for Clinical and Experimental Ophthalmology*. 2017;255(10):1935-1946.
2. Daniel E, Ying GS, Siatkowski RM, et al. Intraocular Hemorrhages and Retinopathy of Prematurity in the Telemedicine Approaches to Evaluating Acute-Phase Retinopathy of Prematurity (e-ROP) Study. *Ophthalmology*. Mar 2017;124(3):374-381.
3. Hutcheson KA, Nguyen ATQ, Preslan MW, Ellish NJ, Steidl SM. Vitreous hemorrhage in patients with high-risk retinopathy of prematurity. *American Journal of Ophthalmology*. 2003;136(2):258-263.
4. Campbell JP, Ataer-Cansizoglu E, Bolon-Canedo V, et al. Expert Diagnosis of Plus Disease in Retinopathy of Prematurity From Computer-Based Image Analysis. *JAMA Ophthalmol*. Jun 1 2016;134(6):651-7.
5. Mao J, Luo Y, Liu L, et al. Automated diagnosis and quantitative analysis of plus disease in retinopathy of prematurity based on deep convolutional neural networks. *Acta Ophthalmol*. Sep 27 2019;
6. Hewing NJ, Kaufman DR, Chan RV, Chiang MF. Plus disease in retinopathy of prematurity: qualitative analysis of diagnostic process by experts. *JAMA Ophthalmol*. Aug 2013;131(8):1026-32.
7. Huang G, Liu Z, Van Der Maaten L, Weinberger KQ. Densely connected convolutional networks. https://arxiv.org/abs/1608.06993 ArXiv 2016. Accessed September 16, 2018.
8. Hubbard GB, 3rd. Surgical management of retinopathy of prematurity. *Curr Opin Ophthalmol*. Sep 2008;19(5):384-90.
9. Guo S, Yan J, Yang T, Yang X, Bezard E, Zhao B. Protective effects of green tea polyphenols in the 6-OHDA rat model of Parkinson's disease through inhibition of ROS-NO pathway. *Biol Psychiatry*. Dec 15 2007;62(12):1353-62.
10. Sandler M, Howard A, Zhu M, Zhmoginov A, Chen L-C. MobileNetV2: Inverted Residuals and Linear Bottlenecks. https://arxiv.org/abs/1801.04381 ArXiv. 2018. Accessed February 25, 2019.
11. Tan M, Chen B, Pang R, et al. MnasNet: Platform-Aware Neural Architecture Search for Mobile. https://arxiv.org/abs/1807.11626 ArXiv. 2018. Accessed May 15, 2019.
12. Szegedy C, Vanhoucke V, Ioffe S, Shlens J. Rethinking the Inception Architecture for Computer Vision. https://arxiv.org/abs/1512.00567 ArXiv. 2016. Accessed January 6, 2020.
13. Chollet F. Xception: Deep Learning with Depthwise Separable Convolutions https://arxiv.org/abs/1610.02357 ArXiv. 2016. Accessed January 20, 2020.
14. Szegedy C, Ioffe S, Vanhoucke V, Alemi A. Inception-v4, Inception-ResNet and the Impact of Residual Connections on learning. https://arxiv.org/abs/1602.07261 ArXiv. 2016. Accessed August 30, 2019.
15. Minaee S, Boykov Y, Porikli F, Plaza A, Kehtarnavaz N, Terzopoulos D. Image Segmentation Using Deep Learning: A Survey. https://arxiv.org/abs/2001.05566 ArXiv. 2020. Accessed May 6, 2020.
16. Raghu M, Zhang C, Kleinberg J, Bengio S. Transfusion: Understanding Transfer Learning for Medical Imaging. https://arxiv.org/abs/1902.07208 ArXiv. 2019. Accessed July 16, 2020.
17. Kingma DP, Ba JL. ADAM: a method for stochastic potimization. https://arxiv.org/abs/1412.6980 ArXiv. 2015. Accessed July 15, 2020.
18. Zhang MR, Lucas J, Hinton G, Ba J. Lookahead Optimizer: k steps forward, 1 step back. https://arxiv.org/abs/1907.08610 ArXiv. 2019. Accessed June 18, 2020.
19. Guo C, Pleiss G, Sun Y, Weinberger KQ. On Calibration of Modern Neural Networks. https://arxiv.org/pdf/1706.04599.pdf ArXiv. 2017. Accessed June 18, 2020.

© 2021 Wang J et al. *JAMA Network Open*.

20. Ju C, Bibaut A, van der Laan M. The Relative Performance of Ensemble Methods with Deep Convolutional Neural Networks for Image Classification. *J Appl Stat*. 2018;45(15):2800-2818.
21. Lundberg S, Lee S-I. A Unified Approach to Interpreting Model Predictions. https://arxiv.org/abs/1705.07874 ArXiv. 2017. Accessed January 3, 2020.
22. Zhou B, Khosla A, Lapedriza A, Oliva A, Torralba A. Learning Deep Features for Discriminative Localization. https://arxiv.org/abs/1512.04150 ArVix. 2015. Accessed February 27, 2020.
23. Selvaraju RR, Cogswell M, Das A, Vedantam R, Parikh D, Batra D. Grad-CAM: Visual Explanations from Deep Networks via Gradient-based Localization. https://arxiv.org/abs/1610.02391 ArXiv. 2016. Accessed April 5, 2020.
24. Simonyan K, Vedaldi A, Zisserman A. Deep inside convolutional networks: visualising image classification models and saliency maps. https://arxiv.org/abs/1312.6034 ArXiv. 2013. Accessed May 16, 2020.
25. Sundararajan M, Taly A, Yan Q. Axiomatic attribution for deep networks. https://arxiv.org/abs/1703.01365 ArXiv. 2017. Accessed May 30, 2020.
26. Bach S, Binder A, Montavon G, Klauschen F, Müller K-R, Samek W. On Pixel-Wise Explanations for Non-Linear Classifier Decisions by Layer-Wise Relevance Propagation. *PLOS ONE*. 2015;10(7):e0130140.
27. Fabian Isensee, Jens Petersen, Andre Klein, et al. nnU-Net: Self-adapting Framework for U-Net-Based Medical Image Segmentation. https://arxiv.org/abs/1809.10486 ArXiv. 2018. Accessed June 18, 2020.
28. Fausto M, Nassir N, Seyed-Ahmad A. V-Net: Fully Convolutional Neural Networks for Volumetric Medical Image Segmentation. https://arxiv.org/abs/1606.04797 ArXiv. 2016. Accessed June 06, 2020.
